# Immune Checkpoint Axes Are Dysregulated in Patients With Alcoholic Hepatitis

**DOI:** 10.1002/hep4.1475

**Published:** 2020-01-12

**Authors:** Wei Li, Ying Xia, Jing Yang, Haitao Guo, Guoqing Sun, Arun J. Sanyal, Vijay H. Shah, Yongliang Lou, Xiaoqun Zheng, Naga Chalasani, Qigui Yu

**Affiliations:** ^1^ Department of Microbiology and Immunology Indiana University School of Medicine Indianapolis IN; ^2^ Department of Clinical Laboratory the Second Affiliated Hospital and Yuying Children’s Hospital of Wenzhou Medical University Wenzhou Zhejiang China; ^3^ School of Laboratory Medicine Wenzhou Medical University Wenzhou China; ^4^ Division of Gastroenterology and Hepatology Department of Medicine Virginia Commonwealth University Richmond VA; ^5^ Division of Gastroenterology and Hepatology Mayo Clinic Rochester MN; ^6^ Division of Gastroenterology and Hepatology Department of Medicine Indiana University School of Medicine Indianapolis IN

## Abstract

Alcoholic hepatitis (AH) is a severe inflammatory liver disease that develops in some heavy drinkers. The immune system in patients with AH is hyperactive and yet dysfunctional. Here, we investigated whether this immune‐dysregulated state is related to the alcoholic impact on immune checkpoints (ICPs). We used multiplex immunoassays and enzyme‐linked immunosorbent assay to quantify plasma levels of 18 soluble ICPs (sICPs) from 81 patients with AH, 65 heavy drinkers without liver diseases (HDCs), and 39 healthy controls (HCs) at baseline, 33 patients with AH and 32 HDCs at 6‐month follow‐up, and 18 patients with AH and 29 HDCs at 12‐month follow‐up. We demonstrated that baseline levels of 6 sICPs (soluble T‐cell immunoglobulin and mucin domain 3 [sTIM‐3], soluble cluster of differentiation [sCD]27, sCD40, soluble Toll‐like receptor‐2 [sTLR‐2], soluble herpesvirus entry mediator [sHVEM], and soluble lymphotoxin‐like inducible protein that competes with glycoprotein D for herpes virus entry on T cells [sLIGHT]) were up‐regulated, while 11 sICPs (soluble B‐ and T‐lymphocyte attenuator [sBTLA], sCD160, soluble cytotoxic T‐lymphocyte‐associated protein 4 [sCTLA‐4], soluble lymphocyte‐activation gene 3 [sLAG‐3], soluble programmed death 1 [sPD‐1], sPD ligand 1 [sPD‐L1], sCD28, soluble glucocorticoid‐induced tumor necrosis factor receptor‐related protein [sGITR], sGITR ligand [sGITRL], sCD80, and inducible T‐cell costimulator [sICOS]) were down‐regulated in patients with AH compared to HDCs. The up‐regulated sICPs except sLIGHT and down‐regulated sCD80, sCD160, sCTLA‐4, and sLAG‐3 correlated positively or negatively with AH disease severity, bacterial translocation, and inflammatory factors. At follow‐up, abstinent patients with AH still had higher levels of several sICPs compared to HDCs. We also compared expression of 10 membrane‐bound ICPs (mICPs) on peripheral blood mononuclear cells (PBMCs) from patients with AH and HCs by flow cytometry and found that several mICPs were dysregulated on blood cells from patients with AH. The function and regulation of sICPs and mICPs were studied using PBMCs from patients with AH and HCs. Recombinant sHVEM affected tumor necrosis factor (TNF)‐α and interferon‐γ production by T cells from patients with AH and HCs. *Conclusion:* Both sICPs and mICPs were dysregulated in patients with AH, and alcohol abstinence did not fully reverse these abnormalities. The HVEM axis plays a role in regulating T‐cell function in patients with AH.

## Graphical abstract

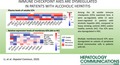

**Abbreviations**
AH: alcoholic hepatitis
ALT: alanine aminotransferase
APC: antigen‐presenting cell
AST: aspartate aminotransferase
BTLA: B‐ and T‐lymphocyte attenuator
CD: cluster of differentiation
CRP: C‐reactive protein
CTLA‐4: cytotoxic T‐lymphocyte‐associated protein 4
DMSO: dimethyl sulfoxide
ELISA: enzyme‐linked immunosorbent assay
GITR: glucocorticoid‐induced tumor necrosis factor receptor‐related protein
GITRL: glucocorticoid‐induced tumor necrosis factor receptor‐ligand
HC: healthy control
HDC: heavy drinking control
HVEM: herpesvirus entry mediator
HVEM‐his: his‐tagged recombinant human herpesvirus entry mediator
ICOS: inducible T‐cell costimulator
ICP: immune checkpoint
IFN: interferon
IgG: immunoglobulin G
IL: interleukin
LAG‐3: lymphocyte‐activation gene 3
LBP: lipopolysaccharide‐binding protein
LIGHT: lymphotoxin‐like inducible protein that competes with glycoprotein D for herpes virus entry on T cells
LPS: lipopolysaccharide
LT‐α: lymphotoxin‐alpha
m: membrane bound
mDF: Maddrey’s discriminant function
MELD: Model for End‐Stage Liver Disease
MMP: matrix metalloproteinase
NK cell: natural killer
NKT: cell natural killer T cell
ns: not significant
PBMC: peripheral blood mononuclear cell
PBS: phosphate‐buffered saline
PD‐1: programmed death 1
PD‐L1: programmed death ligand 1
s: soluble
TCR: T‐cell receptor
TIM‐3: T‐cell immunoglobulin and mucin domain 3
TLR‐2: Toll‐like receptor 2
TNF: tumor necrosis factor
TREAT: Translational Research and Evolving Alcoholic Hepatitis Treatment

Alcoholic hepatitis (AH) is a severe inflammatory liver disease that develops in 10%‐35% of chronic heavy drinkers. Although the exact trigger for development of AH is still unclear, alcohol‐induced translocation of gut bacteria and bacterial components into the liver and blood and subsequent activation of liver‐resident macrophages (Kupffer cells) and other immune and nonimmune cells play a critical role in the initiation and progression of AH.^1^, ^2^, ^3^ Patients with AH have elevated levels of a wide range of proinflammatory factors, such as interleukin‐8 (IL‐8) and tumor necrosis factor alpha (TNF‐α), and their immune cells are highly dysregulated, which is characterized by immune hyperactivation, exhaustion, and dysfunction.^1^, ^3^, ^4^, ^5^, ^6^

Immune homeostasis, which is critical for maintaining immune self‐tolerance and preventing overexuberant immune responses, is regulated by multiple factors, including balanced signals from a network of immune costimulatory and coinhibitory receptors/ligands, collectively known as immune checkpoints (ICPs). More than 20 ICP pathways consisting of ICP receptors and their ligands have been identified, such as the costimulatory cluster of differentiation (CD)28/CD80/CD86 pathway and the inhibitory pathways of cytotoxic T‐lymphocyte‐associated protein 4 (CTLA‐4)/CD80/CD86 and programmed death 1 (PD‐1)/PD ligand 1 (PD‐L1)/PD‐L2. Among the ICPs, herpesvirus entry mediator (HVEM) serves as a shared receptor or ligand for stimulatory and inhibitory ligands/receptors, including lymphotoxin‐alpha (LT‐α), lymphotoxin‐like inducible protein that competes with glycoprotein D for herpes virus entry on T cells (LIGHT), B‐ and T‐lymphocyte attenuator (BTLA), and CD160. HVEM and its ligands are expressed on both hematopoietic and nonhematopoietic cells. Ligation of HVEM with LT‐α, LIGHT, BTLA, or CD160 stimulates immune responses, while as a ligand, HVEM triggers inhibitory signaling in BTLA^+^ and CD160^+^ T cells but stimulatory signaling in CD160^+^ natural killer (NK) cells.^7^, ^8^ Thus, HVEM mediates bidirectional signaling and serves as a molecular switch between stimulatory and inhibitory signaling, thereby playing a unique role in immune homeostasis. Currently, the regulation and function of HVEM in AH pathogenesis is not known.

Different ICPs are expressed at different levels and at distinct checkpoints to fine‐tune immune responses. However, chronic inflammation in the settings of cancer, autoimmunity, chronic infection, and sepsis leads to persistent hyperexpression of multiple coinhibitory ICPs and subsequent functional paralysis/exhaustion of the immune system. Blockade of the immunosuppressive CTLA‐4, PD‐1, and PD‐L1 pathways with ICP inhibitors has been successfully used to restore and enhance the antitumor activity of cytotoxic T lymphocytes.^9^, ^10^ Targeting the main inhibitory ICPs (CTLA‐4, lymphocyte‐activation gene 3 [LAG‐3], PD‐1, and T‐cell immunoglobulin and mucin domain 3 [TIM‐3]) pathways has also been explored for therapeutic intervention in inflammatory diseases.^11^, ^12^ Recently, *ex vivo* blockage of PD‐1 and TIM‐3 has been shown to restore functions of immune cells from patients with AH.^6^

ICP molecules also exist in soluble forms. Similar to membrane‐bound ICPs (mICPs), soluble ICPs (sICPs) are also present in normal physiologic conditions and are highly dysregulated in patients with cancer or various inflammatory diseases.^12^, ^13^, ^14^, ^15^, ^16^ Soluble ICPs can be generated through either alternative messenger RNA (mRNA) splicing and secretion, extracellular release of membrane‐bound receptors from exosomes, or protease‐mediated shedding from mICPs by actions of matrix metalloproteinases (MMPs). Some, if not all, sICPs exhibit biological functions as decoy receptors/ligands for mICPs or as agonists to positively or negatively regulate immune responses.^16^, ^17^, ^18^ Levels of circulating sICPs are often elevated in cancer and inflammatory diseases, serving as promising prognostic and/or predictive biomarkers.^16^

Currently, there are no systematic studies of either mICPs or sICPs in patients with AH. Given that ICPs are the key regulators of immune responses and that patients with AH are in a highly immune‐dysregulated state, ICP axes are likely dysregulated in patients with AH. To this end, we performed comprehensive studies of mICPs and sICPs in the peripheral blood of a large cohort of patients with AH and matched heavy drinkers without liver disease (HDCs) as well as healthy controls (HCs). We also studied the effects of long‐term alcohol abstinence on the regulation of ICPs and the relationships of ICP profiles with patient clinical parameters and plasma levels of bacterial translocation‐associated markers, inflammatory cytokines/chemokines, and MMPs. In addition, we used peripheral blood cells to study the mechanisms underlying the molecular origin, regulation, and function of ICPs. We found that both sICPs and mICPs were highly dysregulated in patients with AH and that alcohol abstinence did not fully reverse these abnormalities.

## Participants and Methods

### Human Subjects and Blood Samples

The subjects with heavy alcohol consumption (81 patients with AH and 65 HDCs at baseline, 33 patients with AH and 32 HDCs at 6‐month follow‐up, and 18 patients with AH and 29 HDCs at 12‐month follow‐up) were part of the multicenter prospective Translational Research and Evolving Alcoholic Hepatitis Treatment (TREAT) consortium (https://clinicaltrials.gov/ct2/show/NCT02172898), as described in our previous studies.^4^, ^19^ HDCs were individuals with a comparable history of alcohol consumption but had no overt evidence of liver disease. Thirty‐nine healthy volunteers were included as HCs. Peripheral blood was obtained from these study subjects and separated into plasma and peripheral blood mononuclear cells (PBMCs). 

This study was approved by the Institutional Review Boards at Indiana University School of Medicine, Mayo Clinic, and Virginia Commonwealth University. All participants provided written informed consent.

### Multiplex Immunoassays

The Human Immuno‐Oncology Checkpoint Protein Panel (MilliporeSigma, Burlington, MA) was used to simultaneously measure plasma concentrations of 16 sICPs (sBTLA, sCD27, sCD28, sCD40, sCD80/B7‐1, sCD86/B7‐2, sCTLA‐4, soluble glucocorticoid‐induced tumor necrosis factor receptor‐related protein (sGITR), sGITR ligand (sGITRL), sHVEM, soluble inducible T‐cell costimulator (sICOS), sLAG‐3, sPD‐1, sPD‐L1, sTIM‐3, and soluble Toll‐like receptor 2 [sTLR‐2]). Human MMP Magnetic Panel 2 (MilliporeSigma) was used to simultaneously quantify plasma levels of five MMPs, including MMP1, MMP2, MMP7, MMP9, and MMP10.

### Enzyme‐Linked Immunosorbent Assays

Plasma levels of sCD160 and sLIGHT were quantified using the Human CD160 Matched enzyme‐linked immunosorbent assay (ELISA) Antibody Pair Set (Sino Biological, Beijing, China) and the Human LIGHT Duoset ELISA Kit (R&D Systems, Minneapolis, MN), respectively. Plasma levels of the endotoxin lipopolysaccharide (LPS), soluble LPS‐binding protein (sLBP), soluble CD14 (sCD14), and soluble CD163 (sCD163) were used as surrogate markers for bacterial translocation from the gut to the systemic circulation. LPS amounts were determined as described.^4^ Plasma levels of sLBP, sCD14, and sCD163 were quantified using the Human LBP DuoSet ELISA kit and the Human CD14 and CD163 Quantikine kits (R&D Systems), respectively. Levels of sHVEM in cell‐free supernatant of *in vitro* monocyte cultures were determined using the Human HVEM DuoSet ELISA kit (R&D Systems).

### Cell Culture and *In Vitro* Stimulation

To determine the effect of LPS and alcohol on sHVEM production and mHVEM expression on the surface of monocytes and B cells, 0.4 × 10^6^ PBMCs or purified monocytes using Human CD14 Microbeads (Miltenyi Biotec, Auburn, CA) from HCs were treated with 200 ng/mL LPS and/or 250 mM ethanol for 24 hours. Cell‐free supernatant and cells were used for ELISA and flow cytometric analysis to determine the expression of sHVEM and mHVEM, respectively.

### MMP Cleavage and Biological Function of sHVEM

To test the effect of MMPs on shedding of HVEM from monocytes, purified CD14^+^ monocytes were pretreated with GM6001 (MilliporeSigma), a broad‐spectrum MMP inhibitor, at 20 μM or dimethyl sulfoxide (DMSO) as the vehicle control for 30 minutes, followed by LPS and alcohol treatment for 24 hours. The expressions of sHVEM and mHVEM were measured as described above.

To test the effect of sHVEM on T‐cell function, 0.2 × 10^6^ PBMCs from patients with AH and HCs were pretreated with 5 μg/mL his‐tagged recombinant human HVEM (HVEM‐his; Sino Biological) or phosphate‐buffered saline (PBS) for 30 minutes at 37°C, followed by treatment with soluble anti‐CD3/anti‐CD28 antibodies (1 μg/mL each; both from Biolegend, Diego, CA) for 20 hours. Brefeldin A was added for the last 6 hours of stimulation. Cells were subjected to intracellular staining (ICS) of cytokines. PBMCs were also pretreated with 5 μg/mL recombinant human HVEM‐Fc fusion protein (R&D Systems) or human immunoglobulin G‐1 (hIgG1) control (BioLegend; San Diego, CA) before stimulation with anti‐CD3/anti‐CD28 antibodies.

### Flow Cytometric Analysis

To stain mICPs on the subsets of peripheral blood immune cells, PBMCs from patients with AH and HCs were incubated with fixable viability dye (Thermo Fisher Scientific, Cincinnati, OH), followed by staining with fluorochrome‐conjugated antibodies against cell lineage markers (CD3, CD4, CD14, CD19, and CD56) and 10 ICPs (BTLA, CD27, CD40, CD160, CTLA‐4, HVEM, LAG‐3, LIGHT, PD‐1, and TIM‐3). All antibodies were from BioLegend.

For ICS, PBMCs were stained with fixable viability dye, followed by fluorochrome‐conjugated antibodies against human CD4 and CD8. Subsequently, cells were fixed and permeabilized with the Cytofix/Cytoperm reagents (BD Biosciences, San Jose, CA) and stained with antibodies against TNF‐α and interferon‐gamma (IFN‐γ) (BioLegend).

### Statistical Analysis

Chi‐square test was used for comparison between groups for categorical variables. Mann‐Whitney test and Kruskal‐Wallis test with Dunn’s corrections were used to calculate differences in continuous variables between two groups and among three groups in cross‐sectional analysis, respectively. The linear relationship between ICPs and clinical or inflammatory factors was analyzed using the Spearman correlation test. Friedman rank sum test with Dunn’s corrections was used to calculate differences in longitudinal analysis. Cox regression analysis was performed to identify sICPs associated with 90‐day mortality. Soluble ICPs with *P* < 0.10 in univariate analysis were entered into a multivariate model using the backward elimination method to identify independent predictors of 90‐day mortality. Repeated measures analysis of variance test followed by Dunnett’s test was used to compare differences in levels of membrane and soluble HVEM between the medium group and other *in vitro* treatment groups. Paired *t* test was used to compare differences in membrane and soluble HVEM between DMSO and GM6001 groups and to compare differences in cytokine production between HVEM‐his and PBS groups and between HVEM‐Fc and hIgG1. *P* < 0.05 was considered statistically significant.

## Results

### Characteristics of the Study Cohort

The demographic and clinical characteristics of the study subjects are summarized in Table 1. Age and sex distributions were similar among patients with AH, HDCs, and HCs at baseline and between patients with AH and HDCs at follow‐ups. HDCs had significantly more drinks than patients with AH in the last 30 days before the enrollment and at day 360. Although baseline total bilirubin and creatinine were not different between HDCs and HCs, both aspartate aminotransferase (AST) and alanine aminotransferase (ALT) levels were higher in HDCs than HCs. At enrollment and follow‐up, patients with AH had higher Model for End‐Stage Liver Disease (MELD) scores, liver biochemistries (AST, ALT, and total bilirubin), and longer prothrombin time than HDCs. Forty‐eight patients with AH (59%) were treated with prednisone alone (n = 33), pentoxifylline alone (n = 2), or both drugs (n = 13); 14 patients with AH died within 3 months after enrollment. At follow‐up, patients with AH and HDCs drank much less and liver biochemistries greatly improved but were not normalized in patients with AH and remained unchanged for HDCs. For patients who achieved complete alcohol abstinence at follow‐up, MELD score, total bilirubin, AST, and prothrombin time were still higher in patients with AH than HDCs (Supporting Table S1).

**Table 1:** Characteristics of Patients with AH and HDCs in the TREAT Study Cohort

Variable	Day 0 (Baseline)				Day 180			Day 360		
	HC (n = 39)	AH (n = 81)	HDC (n = 65)	*P*	AH (n = 33)	HDC (n = 32)	*P*	AH (n = 18)	HDC (n = 29)	*P*
Age (years)	42 (26‐52)	45 (35‐52)	47 (37‐54)	ns	43 (37‐53)	47 (35‐54)	ns	45 (34‐53)	50 (40‐55)	ns
Sex (% male)	64	57	62	ns	67	66	ns	61	66	ns
Total drinks in 30 days		191 (68‐330)	261 (190‐481)	†	0 (0‐7)	0 (0‐60)	ns	0 (0‐0.75)	38 (0‐119)	*
Total drinking days in 30 days		26 (14‐30)	28 (23‐30)	ns	0 (0‐2)	0 (0‐16)	ns	0 (0‐0.75)	10 (0‐26)	ns
MELD score		24 (20‐27)	7 (6‐8)	‡	12 (8‐14)	7 (6‐8)	‡	10 (8‐13)	7 (6‐8)	‡
Creatinine (mg/dL)	0.9 (0.8‐1)^a^	0.8 (0.6‐1.1)	0.8 (0.7‐1)	ns	0.7 (0.6‐1)	0.9 (0.8‐1.1)	*	0.9 (0.6‐1.2)	0.9 (0.8‐1.1)	ns
Total bilirubin (mg/dL)	0.3 (0.3‐0.6)^a^	14.4¶ (7.5‐24.2)	0.6 (0.4‐0.7)	‡	1.6 (0.8‐4.2)	0.4 (0.3‐0.5)	‡	1.1 (0.7‐1.8)	0.4 (0.3‐0.7)	‡
AST (IU/L)	17 (15‐20)^a^	106¶ (83‐145)	24§ (18‐31)	‡	48 (32‐71)	20 (16‐23)	‡	33 (26‐57)	22 (17‐38)	†
ALT (IU/L)	11 (8‐17)^a^	46¶ (31‐59)	22# (16‐31)	‡	27 (20‐44)	16 (11‐23)	‡	24 (21‐33)	18 (13‐35)	ns
Prothrombin time (INR)		1.8 (1.5‐2.1)	1.0 (0.9‐1.0)	‡	1.3 (1.2‐1.5)	1.0 (0.9‐1.0)	‡	1.2 (1‐1.5)	1.0 (0.9‐1.0)	‡
Treatment with PDN and/or PTX		59%	0%		52%	0%		56%	0%	

Data are represented as median and (interquartile ranges). Kruskal‐Wallis test with Dunn’s correction for pairwise comparisons of continuous variables among HC, AH patients, and HDC at enrollment (Day 0). Mann Whitney test comparing AH patients versus HDC at day 180 and day 360 follow‐up. Chi‐square test for analysis of categorical variables.

a Data from 33 HC

* *P* < 0.05.

^†^ *P* < 0.01.

^‡^ *P* < 0.001 for comparison between AH patients and HDC.

^§^ *P* < 0.05.

^¶^ *P* < 0.001 for comparison between AH patients and HC at Day 0.

^#^ *P* < 0.001 for comparison between HDC and HC at Day 0.

Abbreviations: AH, patients with alcoholic hepatitis; ALT, alanine aminotransferase; AST, aspartate aminotransferase; HC, healthy controls; HDC, heavy drinking controls; INR, international normalized ratio; MELD, model for end‐stage liver disease; ns, not significant; PDN, prednisone; PTX, pentoxifylline.

### Plasma Levels of Soluble ICPs Were Highly Dysregulated in Patients With AH

Plasma levels of 18 sICPs, including 10 stimulatory, seven inhibitory, and one dual functional ICPs, are summarized in Table 2, Supporting Table S2, and Supporting Fig. S1. All 18 sICPs were detectable in HCs, indicating that they could play biological roles in immune homeostasis under physiologic conditions. Compared to HCs and HDCs, patients with AH had significantly higher baseline levels of two stimulatory sICPs (sCD27 and sCD40), one inhibitory sICP (sTIM‐3), and the bifunctional sHVEM. Patients with AH also had higher baseline levels of sLIGHT and sTLR‐2 than HDCs. Plasma sTIM‐3 was the most highly up‐regulated sICP in patients with AH, followed by sHVEM, sCD40, sCD27, sTLR‐2, and sLIGHT. Baseline levels of the remaining six inhibitory sICPs (sBTLA, sCD160, sCTLA‐4, sLAG‐3, sPD‐1, and sPD‐L1) and five stimulatory sICPs (sCD28, sCD80, sGITR, sGITRL, and sICOS) were down‐regulated in patients with AH, with sICOS and sCD160 among the most down‐regulated. Plasma sCD86 level did not differ among HCs, patients with AH, and HDCs. Interestingly, five sICPs were also dysregulated in HDCs, as sCD40 and sTIM‐3 were up‐regulated while sCD80, sGITRL, and sLIGHT were down‐regulated relative to HCs. These results suggest that plasma levels of these five sICPs (sCD40, sCD80, sGITRL, sICOS, and sTIM‐3) could serve as sensitive markers for subclinical immune activation or dysregulation in HDCs.

**Table 2:** Comparison of Plasma Levels of Soluble ICPs in HCs, Patients With AH, and HDCs

Soluble ICP (pg/mL)		Day 0 (Baseline)			Day 180		Day 360	
		HC (n = 34‐39)	AH (n = 63‐81)	HDC (n = 48‐65)	AH (n = 24‐33)	HDC (n = 29‐32)	AH (n = 16‐18)	HDC (n = 26‐29)
Stimulatory ICPs	CD27	881|| (603‐1,180)	2,079‡ (1,223‐4,263)	1,177 (672‐2,158)	1,963* (891‐3,834)	1,060 (666‐1,788)	1,738 (1,087‐3,183)	1,457 (805‐2,469)
	CD28	1,087¶ (496‐2,106)	666‡ (192‐1,090)	1,287 (789‐1,899)	750 (367‐1,408)	944 (495‐1,635)	944 (432‐1,762)	940 (509‐1,836)
	CD40	285|| (148‐385)	764‡ (554‐1,734)	417# (335‐587)	556‡ (442‐643)	400 (315‐481)	504 (359‐546)	366 (307‐497)
	CD80	21|| (13‐31)	9‡ (5‐10)	14§ (9‐19)	9 (6‐15)	9 (6‐16)	9 (6‐26)	11 (9‐21)
	CD86	718 (457‐1,228)	720 (449‐1,065)	663 (399‐924)	932‡ (665‐1,244)	624 (390‐800)	698 (384‐1,388)	731 (434‐1,150)
	GITR	16¶ (6‐48)	6* (6‐6)	6 (6‐24)	6 (6‐20)	10 (6‐31)	10 (6‐33)	19 (8‐46)
	GITRL	169¶ (81‐427)	48 (16‐283)	109§ (42‐163)	148 (21‐477)	116 (63‐172)	153 (32‐571)	143 (79‐248)
	ICOS	370|| (139‐668)	45‡ (45‐72)	149 (72‐231)	45 (45‐198)	100 (45‐259)	60 (45‐203)	238 (57‐423)
	LIGHT	240 (39‐1,274)	282* (48‐653)	82** (0‐579)	497 (20‐1,919)	196 (0‐1,123)	352 (29‐1,437)	36 (0‐602)
	TLR‐2	466 (286‐935)	670* (393‐900)	505 (297‐671)	481* (336‐471)	306 (239‐474)	388 (194‐829)	499 (264‐697)
Inhibitory ICPs	BTLA	370|| (139‐668)	105† (22‐246)	199 (75‐430)	173 (22‐370)	154 (48‐370)	119 (22‐386)	262 (139‐421)
	CD160	5,590|| (3,801‐10,605)	884‡ (818‐2,520)	8,582 (3,727‐15,229)	5,241 (2,149‐11,635)	10,507 (4,032‐13,454)	8,061 (1,954‐14,671)	7,993 (3,012‐13,565)
	CTLA‐4	31¶ (12‐81)	10‡ (4‐20)	27 (15‐49)	18 (4‐46)	20 (4‐52)	17* (4‐60)	57 (16‐95)
	HVEM	1,197|| (885‐1,651)	3,420‡ (1,848‐4,956)	1,577 (1,209‐2,097)	2,475‡ (1,802‐3,402)	1,569 (1,111‐1,886)	1,787 (1,280‐3,141)	1,639 (1,005‐2,043)
	LAG‐3	3,459|| (2,120‐4,852)	1,601‡ (996‐2,812)	3,128 (1,659‐4,378)	2,771 (1,602‐5,423)	2,391 (1,727‐3,342)	1,902 (1,287‐3,350)	2,481 (1,652‐3,975)
	PD‐1	361 (219‐744)	248* (172‐421)	396 (238‐589)	338 (248‐562)	300 (217‐443)	362 (233‐657)	404 (239‐620)
	PD‐L1	19|| (10‐41)	9‡ (5‐14)	22 (12‐31)	12 (5‐22)	14 (4‐25)	5† (3‐22)	25 (8‐44)
	TIM‐3	1,228|| (1,021‐1,875)	6,340‡ (3,451‐10,374)	1,914§ (1,288‐2,546)	3,239‡ (2,092‐4,430)	1,701 (2,085‐4,740)	2,663† (2,085‐4,740)	1,739 (1,154‐2,611)

Data are represented as median and (interquartile ranges) in pg/mL. Kruskal‐Wallis test with Dunn’s correction for pairwise comparisons among HC, AH patients, and HDC at baseline (Day 0). Mann Whitney test comparing AH patients versus HDC at day 180 and day 360 follow‐ups.

* *P* < 0.05.

^†^ *P* < 0.01.

^‡^ *P* < 0.001 for comparison between AH patients and HDC.

^§^ *P* < 0.05.

^||^ *P* < 0.01.

^¶^ *P* < 0.001 for comparison between AH patients and HC.

^#^ *P* < 0.001 for comparison between HDC and HC.

** *P* < 0.01.

Abbreviations: AH, patients with alcoholic hepatitis; HC, healthy controls; HDC, heavy drinking controls; ICPs, immune checkpoints.

At follow‐up, plasma levels of sICPs tended to be normalized in patients with AH but remained unchanged in HDCs. However, at 6‐month follow‐up, five out of the six up‐regulated sICPs (sCD27, sCD40, sHVEM, sTIM‐3, and sTLR‐2) were still elevated in patients with AH whereas all the 11 down‐regulated sICPs became similarly expressed between patients with AH and HDCs. At 12‐month follow‐up, only sTIM‐3 remained higher while sCTLA‐4 and sPD‐L1 were still lower in patients with AH compared to HDCs (Table 2; Supporting Table S2). Together, our data indicate that plasma levels of sICPs were highly dysregulated in patients with AH and that there was an incomplete reversal of those abnormalities at follow‐up.

### Association Between Plasma Levels of Soluble ICPs and Clinical Parameters in Patients With AH

Five up‐regulated sICPs (sCD27, sCD40, sHVEM, sTIM‐3, and sTLR‐2) positively correlated with the MELD score, and some also had positive correlations with total bilirubin, creatinine, AST, or the systematic inflammation marker c‐reactive protein (CRP) (Table 3). On the other hand, among the 11 down‐regulated sICPs, four (sCD80, sCD160, sCTLA‐4, and sLAG‐3) correlated inversely with MELD score and total bilirubin. Plasma levels of sCD80, sCD160, and sCTLA‐4 also had negative correlations with creatinine, prothrombin time, or ALT (Table 3). We also analyzed associations between the sICPs and Maddrey’s discriminant function (mDF) score. Soluble CD40 positively correlated (*r* = 0.30, *P* = 0.007) whereas sCD160 (*r* = −0.40, *P* = 0.001) and sCTLA‐4 (*r* = −0.25, *P* = 0.03) negatively correlated with mDF score.

**Table 3:** Correlations of Soluble ICPs With Clinical Parameters, Inflammatory Cytokines, and MMPs in Patients With AH

Variable		Up‐Regulated Soluble ICPs					Down‐Regulated Soluble ICPs			
		CD27	CD40	HVEM	TIM‐3	TLR‐2	CD80	CD160	CTLA‐4	LAG‐3
Clinical parameters	MELD score	0.29*	0.42‡	0.36†	0.27*	0.23*	–0.33†	–0.35†	–0.31†	–0.25*
	Total Bilirubin		0.44‡	0.43‡	0.36‡		–0.36†	–0.28*	–0.29†	–0.37†
	Creatinine	0.43‡	0.62‡	0.42‡	0.29*		–0.32†			
	INR							–0.46‡	–0.25*	
	AST		0.25*			0.27*				
	ALT								0.27*	
	CRP	0.38†	0.45‡		0.41‡	0.28*				
	mDF score		0.30†					–0.40†	–0.25*	
BT markers	LBP								–0.25*	
	LPS	0.29*	0.23*					–0.42†		
	CD14			0.32*	0.27*					
	CD163		0.45‡	0.33†	0.34†	0.36†		–0.28*		
Cytokines/chemokines	IL‐4									
	IL‐6									–0.25*
	IL‐7					–0.32*		0.3*		
	IL‐8	0.28*	0.51‡	0.48‡	0.42‡	0.31*	–0.26*	–0.34*		
	IL‐9									
	IL‐10									
	IL‐15									–0.25*
	IP10				0.28*	0.34†				
	TGF‐α					0.32*				
	TNF‐α									–0.32*
MMPs	MMP1			0.27*						–0.31*
	MMP2				0.41‡			–0.31*		
	MMP7	0.28*	0.57‡	0.44‡		0.31*	–0.26*			–0.32*
	MMP9									
	MMP10	0.33†	0.39†							

The numbers represent Spearman’s coefficients. Negative numbers represent negative correlations.

* *P* < 0.05.

^†^ *P* < 0.01.

^‡^ *P* < 0.001.

Abbreviations: AH, alcoholic hepatitis; ALT, alanine aminotransferase; AST, aspartate aminotransferase; BT, bacterial translocation; CRP, c‐reactive protein; ICPs, immune checkpoints; INR, international normalized ratio for prothrombin time; IP10, interferon‐gamma inducible protein 10; LBP, LPS‐binding protein; MDC, macrophage‐derived chemokine; mDF, Maddrey’s discriminant function; MELD, model for end‐stage liver disease; MMPs, matrix metalloproteinases; TGF, transforming growth factor; TIMPs, tissue inhibitors of metalloproteinases.

We next compared baseline plasma levels of the 18 sICPs in patients with AH who died versus survivors. The nonsurvivors had higher levels of sCD27 and sCD40 and a lower level of sGITRL compared with survivors (Supporting Fig. S2A‐C). In addition, sBTLA was detected in a higher portion of patients with AH who survived in comparison with the nonsurvivors (Supporting Fig. S2D). Next, we performed Cox regression for survival analysis to determine whether any of the sICPs were associated with 90‐day mortality (Supporting Table S3). Univariate analysis revealed that sCD40, sCD28, mDF, and MELD scores and CRP were significantly associated with 90‐day mortality. Multivariate analysis indicated that a high sCD40 level and low levels of sCD28 and sCD160 could predict 90‐day mortality independently of the clinical parameters and therefore might serve as a prognostic marker for AH.

We then determined the effect of prednisone/pentoxifylline treatment on sICP expression in patients with AH (Supporting Table S4). The majority of sICPs were similarly expressed between the treated and untreated patients at recruitment. However, among the six up‐regulated sICPs, sTIM‐3 and sCD40 levels were significantly higher whereas the down‐regulated sGITR, sGITRL, and sCD160 were significantly lower in treated patients. The heightened dysregulation was associated with greater disease severity in treated patients. At 6‐month follow‐up, levels of the up‐regulated sICPs (sTIM‐3, sHVEM, and sCD27) were higher in treated patients with AH. At 12‐month follow‐up, there were no differences in sICP levels between the treated and untreated patients with AH who were abstinent. Our data suggest that steroids/pentoxifylline treatment did not appear to have long‐term immunologic benefits.

### Correlations Between Plasma Levels of Soluble ICPs and Bacterial Translocation Markers and MMPs in Patients With AH

Plasma levels of LPS and several other markers that are associated with bacterial translocation, including LBP, sCD14, and sCD163, are shown in Supporting Tables S5 and S6. Baseline LPS, LBP, sCD14, and sCD163 levels were all elevated in patients with AH in comparison to HDCs and HCs. At follow‐up, levels of those markers greatly decreased from the baseline values in patients with AH while remaining unchanged in HDCs, but sCD163 level remained higher in patients with AH, suggesting that other persistent inflammatory factors, such as TNF‐α,^19^ might play a role in driving sCD163 abnormalities at follow‐up.^20^ We next determined their correlations with the nine sICPs that were associated with liver‐disease severity. LPS levels had positive correlations with sCD27 and sCD40 and negative correlations with sCD160. Plasma sCD14 level positively correlated with sHVEM and sTIM‐3, and sCD163 showed positive correlations with sCD40, sHVEM, sTIM‐3, and sTLR‐2 and a negative correlation with sCD160 (Table 3).

Cytokines are known to regulate expression of ICPs, such as HVEM, PD‐1, and TIM‐3.^21^ We therefore calculated the correlations between levels of 10 cytokines/chemokines that are up‐regulated in patients with AH^19^ with the nine sICPs (Table 3). Only IL‐8 exhibited strong positive correlations with all five up‐regulated sICPs and inverse correlations with sCD80 and sCD160. IFN‐γ inducible protein 10 (IP10) and transforming growth factor alpha (TGF‐α) also had correlations with sTIM‐3 and/or sTLR‐2, while IL‐7 had a negative correlation with up‐regulated TLR‐2 and a positive correlation with the down‐regulated sCD160.

MMPs mediate shedding of extracellular domains of membrane ICPs.^22^ We quantified plasma levels of five major MMPs (MMP1, MMP2, MMP7, MMP9, and MMP10) (Supporting Tables S3 and S4). Compared to HCs and HDCs, patients with AH had significantly higher baseline levels of MMP1, MMP2, MMP7, and MMP10. In addition, patients with AH had a higher level of MMP9 than HCs. At follow‐up, levels of MMP1 and MMP9 were similar between patients with AH and HDCs; however, levels of MMP2, MMP7, and MMP10 remained elevated in patients with AH. To determine whether these up‐regulated MMPs might be involved in sICP production, we analyzed correlations between baseline levels of MMPs and sICPs in patients with AH. As shown in Table 3, sCD27, sCD40, sHVEM, and sTLR‐2 strongly correlated with MMP7, whereas sTIM‐3 had a good correlation with MMP2. Additionally, MMP1 and MMP10 also correlated with sHVEM, sCD27, or sCD40. Together, our data indicated that nine of the 17 dysregulated sICPs correlated with disease severity and markers of bacterial translocation. Highly up‐regulated IL‐8 and multiple MMPs, especially MMP7, were linked to dysregulation of sICPs in patients with AH.

### Dysregulation in Soluble ICPs Was Not Fully Reversed in Patients With AH by Alcohol Abstinence

To determine the impact of alcohol abstinence on recovery of sICP abnormalities, we performed cross‐sectional analysis on follow‐up samples between abstinent patients with AH and HDCs. As shown in Table 4, all 11 down‐regulated sICPs became similarly expressed in patients with AH and HDCs at follow‐up. In contrast, four of the six up‐regulated sICPs (sCD27, sCD40, sHVEM, and sTIM‐3) remained elevated in patients with AH. Next, we performed longitudinal analysis of sICP levels in patients with AH and HDCs who were completely abstinent at follow‐up. None of the six up‐regulated sICPs in patients with AH showed significant reduction throughout the study, although patients with higher baseline levels tended to have decreased production (Supporting Fig. S3A; data not shown). In contrast, four of the 11 down‐regulated sICPs (sBTLA, sCD160, sCD28, and sPD‐L1) were significantly increased at 6‐month or 12‐month follow‐up (Supporting Figs. S3B,C and S4). For HDCs, the 18 sICPs did not show significant changes throughout the study (Supporting Figs. S3 and S4; data not shown). Taken together, our data indicate that the dysregulated sICPs recovered greatly but not completely in abstinent patients with AH.

**Table 4:** Comparison of Plasma Levels of Soluble ICPs in Abstinent Subjects at Follow‐Up on Day 180 and Day 360

Variables		Day 180			Day 360		
		AH (n = 20)	HDC (n = 17)	*P*	AH (n = 14)	HDC (n = 13)	*P*
Up‐regulated soluble ICPs	CD27	2,029 (1,156‐6,568)	816 (642‐1,218)	†	1,784 (1,204‐4,273)	1,138 (606‐1,836)	*
	CD40	554 (451‐631)	373 (285‐473)	‡	506 (359‐563)	312 (291‐435)	*
	HVEM	2,742 (1,875‐3,914)	1,396 (1,034‐1,743)	‡	2,027 (1,327‐3,443)	1,636 (860‐1,898)	*
	LIGHT	503 (24‐938)	85 (10‐1,142)	ns	291 (16‐1,437)	33 (13‐1,003)	ns
	TIM‐3	3,753 (2,043‐4,705)	1,588 (1,251‐2,237)	‡	2,663 (2,000‐4,784)	1,705 (1,139‐2,547)	*
	TLR‐2	435 (282‐650)	374 (276‐470)	ns	388 (194‐1,110)	583 (239‐884)	ns
Down‐regulated soluble ICPs	BTLA	154 (22‐355)	176 (47‐471)	ns	119 (22‐370)	206 (139‐672)	ns
	CD28	779 (535‐1,585)	1,087 (610‐1,670)	ns	944 (326‐1,762)	838 (557‐2,153)	ns
	CD80	9 (9‐19)	9 (5‐17)	ns	9 (5‐20)	11 (9‐21)	ns
	CD160	5,241 (2,098‐13,672)	10,064 (3,557‐12,862)	ns	8,468 (1,634‐15,616)	7,666 (3,287‐10,291)	ns
	CTLA‐4	16 (4‐32)	21 (9‐47)	ns	18 (4‐60)	60 (16‐110)	ns
	GITR	6 (6‐18)	18 (4‐31)	ns	11 (6‐28)	19 (8‐54)	ns
	GITRL	65 (17‐357)	123 (63‐190)	ns	153 (35‐506)	241 (79‐669)	ns
	ICOS	45 (45‐161)	100 (45‐176)	ns	76 (45‐203)	133 (47‐472)	ns
	LAG‐3	2,528 (1,445‐4,224)	2,598 (2,121‐3,430)	ns	2,163 (1,376‐3,350)	2,954 (1,757‐3,975)	ns
	PD‐1	333 (247‐583)	295 (234‐433)	ns	362 (273‐910)	338 (235‐794)	ns
	PD‐L1	10 (5‐22)	15 (7‐25)	ns	10 (3‐22)	22 (10‐90)	ns

Data are represented as median and (interquartile ranges) in pg/mL. Mann Whitney test comparing AH patients vs HDC.

* *P* < 0.05.

^†^ *P* < 0.01.

^‡^ *P* < 0.001.

Abbreviations: AH, patients with alcoholic hepatitis; HDC, heavy drinking controls; ICPs, immune checkpoints; ns, not significant.

### Dysregulated Membrane ICPs in Patients With AH

To determine whether the dysregulated plasma sICPs were linked to expression of mICPs, we compared expression levels of the membrane‐bound counterparts of PD‐1, eight ICPs that were associated with liver damage of patients with AH, and LIGHT on peripheral blood antigen‐presenting cells (APCs; such as monocytes and B cells) and lymphocytes (CD4, CD8, NK, and natural killer T [NKT] cells) from patients with AH versus HCs. The mICPs were grouped as inhibitory/exhaustion (CTLA‐4, LAG‐3, PD‐1, and TIM‐3), stimulatory (CD27 and CD40), and the HVEM axis (HVEM, BTLA, CD160, and LIGHT). Results are summarized in Table 5. The four major inhibitory ICPs (CTLA‐4, LAG‐3, PD‐1, and TIM‐3) were all up‐regulated on CD4 T cells from patients with AH. Expression of LAG‐3 and TIM‐3 were also increased on CD8 T cells and NKT cells. The expression pattern of those inhibitory mICPs on APCs was more complicated. CTLA‐4 and LAG‐3 were up‐regulated on monocytes of patients with AH, while PD‐1 and TIM‐3 were down‐regulated on monocytes. CTLA‐4 was also down‐regulated on B cells from patients with AH. As to the stimulatory mICPs, CD27 was down‐regulated on CD4 T cells while CD40 was also down‐regulated on B cells in patients with AH. The bifunctional HVEM was up‐regulated on all lymphocyte subsets but down‐regulated on APCs (Table 5; Supporting Table S7; Supporting Fig. S5) in patients with AH. BTLA was down‐regulated on B cells but up‐regulated on monocytes and NK cells. CD160 was expressed less on NK cells and B cells. LIGHT was similarly expressed between patients with AH and HCs. Thus, the expression pattern of membrane forms of the HVEM axis and other ICPs on the surface of peripheral blood cells was complex. T cells mainly up‐regulated inhibitory ICPs, whereas monocytes and B cells down‐regulated several ICPs, suggesting that plasma sICPs, including sHVEM, might mainly be derived from shedding of their membrane counterparts from APCs in patients with AH.

**Table 5:** Expression Levels of Membrane ICPs on Subsets of Peripheral Blood Immune Cells From HCs and Subjects with AH

Varibles		Exhaustion/Inhibitory ICPs				Stimulatory ICPs		HVEM Axis			
		CTLA‐4	LAG‐3	PD‐1	TIM‐3	CD27	CD40	HVEM	BTLA	CD160	LIGHT
Mono‐cytes	AH	5,683* (4,891‐7,424)	587† (548‐665)	519‡ (341‐864)	3,659† (3,133‐4,389)	1,337 (1,209‐1,943)	924 (739‐1,070)	10,612* (9,457‐11,457)	14,124* (11,270‐16,565)	1,506 (1,366‐1,831)	618 (382‐691)
	HC	4,443 (4,036‐5,777)	510 (477‐559)	971 (734‐1,235)	5,444 (4,694‐6,729)	1,314 (938‐1,658)	777 (704‐922)	11,622 (10,372‐14,514)	9,632 (8,737‐13,585)	1,427 (1,237‐1,636)	517 (375‐874)
B cells	AH	916* (874‐966)	149 (127‐371)	787 (652‐1,102)	nd	824 (694‐1,154)	3,522† (3,074‐3,979)	9,261† (8,183‐10,376)	10,773* (7,166‐13,527)	458† (168‐554)	179 (122‐262)
	HC	1,050 (962‐1,154)	117 (84‐226)	788 (605‐1,028)	nd	945 (729‐1,034)	4,274 (3,534‐4,603)	11,213 (9,415‐11,664)	14,166 (10,392‐16,982)	746 (644‐982)	152 (73‐216)
NK cells	AH	1,200 (1,015‐1,627)	196 (176‐254)	nd	2,844 (2,183‐3,273)	650 (604‐790)	214 (151‐244)	3,242* (2,867‐3,834)	422‡ (317‐752)	1,956‡ (1,460‐2,648)	276 (146‐413)
	HC	1,102 (1,015‐1,287)	163 (155‐206)	nd	3,122 (2,410‐4,568)	649 (539‐736)	174 (149‐195)	2,770 (2,514‐3,207)	233 (215‐250)	3,033 (2,359‐3,548)	248 (142‐284)
NKT cells	AH	1,024 (802‐1,137)	237* (174‐290)	59 (−127 to 236)	970* (814‐1,456)	1,498 (774‐2,665)	391 (332‐477)	4,358† (3,684‐4,825)	798 (687‐1,006)	991 (715‐1,847)	100 (49‐199)
	HC	890 (686‐1,005)	189 (151‐211)	67 (10‐132)	756 (567‐1,032)	1,128 (974‐1,630)	320 (243‐381)	3,359 (2,790‐3,643)	676 (414‐933)	1,283 (967‐1,743)	133 (−4 to 318)
CD4 T cells	AH	647† (547‐783)	172‡ (152‐180)	297* (233‐339)	715* (628‐1,150)	5,119* (4,455‐6,087)	552 (535‐575)	4,340† (3,453‐4,546)	1,960 (1,698‐2,336)	533 (443‐646)	37 (−84 to 126)
	HC	489 (398‐550)	140 (122‐145)	214 (161‐319)	601 (485‐742)	6,534 (5,398‐7,990)	547 (513‐578)	3,575 (2,978‐3,947)	2,237 (1,817‐2,564)	554 (475‐691)	80 (−125 to 176)
CD8 T cells	AH	915 (829‐1,081)	150* (108‐163)	279 (192‐442)	881‡ (722‐1,050)	4,154 (2,267‐5,098)	235 (196‐264)	3,671* (3,264‐4,216)	1,266 (1,054‐1,455)	731 (595‐1,144)	93 (75‐130)
	HC	791 (617‐859)	103 (80‐137)	254 (215‐367)	684 (607‐737)	3,229 (2,807‐4,927)	207 (191‐222)	3,355 (2,825‐3,639)	1,291 (853.2‐1,655)	998 (653‐1,206)	76 (43‐200)

Data are presented as geometric mean fluorescent intensity. Mann Whitney test was used to compare MFI levels of ICPs expressed on peripheral blood cells of AH patients versus HC. For ICPs except PD‐1 and LIGHT, n = 15‐17 for AH patients; n = 16‐20 for HC. For PD‐1, n = 24 for AH patients; n = 30 for HC. For LIGHT, n = 8 for AH patients; n = 11 for HC.

* *P* < 0.05.

^†^ *P* < 0.01.

^‡^ *P* < 0.001.

Abbrevation: AH, patients with alcoholic hepatitis; HC, healthy controls; ICPs, immune checkpoints; nd, not determined.

### MMP‐Dependent Shedding of HVEM From Monocytes

**Figure 1:**
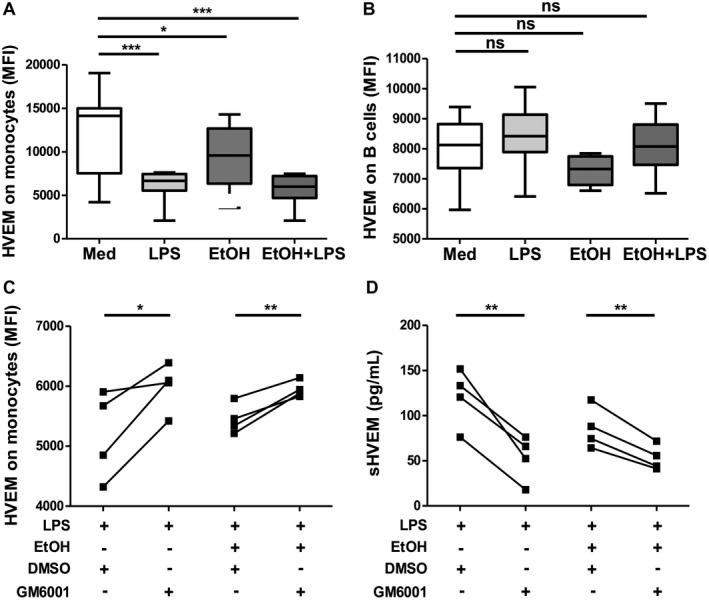
Effects of LPS, ethanol, and MMP on the productions of soluble and membrane forms of ICPs from peripheral immune cells. Box‐and‐whisker graphs showing expression of mHVEM on (A) monocytes and (B) B cells. (C) HVEM expression on monocytes and (D) concentrations of sHVEM in cell‐free supernatant. Purified monocytes of PBMCs from 4 HCs were pretreated with GM6001 or DMSO before incubation with LPS in the presence or absence of ethanol. Repeated measures analysis of variance test followed by Dunnett’s test was used to compare differences between the medium group and other *in vitro* treatment groups. Paired *t* test was used to calculate differences between the DMSO and GM6001 groups. *P < 0.05, **P < 0.01, ***P < 0.001. Graphs show interquartile range (box), median (vertical line), and outliers (whiskers). Abbreviations: EtOH, ethanol; Med, medium; MFI, mean fluorescence intensity.

To explore the mechanisms of HVEM dysregulation on APCs in patients with AH, we treated PBMCs from HCs with LPS in the presence and absence of ethanol. Both LPS and ethanol were able to down‐regulate HVEM expression on the surface of monocytes (Fig. 1A) but did not alter HVEM expression on B cells (Fig. 1B). Next, we examined whether MMPs were involved in LPS‐ and ethanol‐induced down‐regulation of mHVEM on monocytes. Monocytes from HCs were treated with LPS with or without ethanol in the presence of the broad‐spectrum MMP inhibitor GM6001 or DMSO. GM6001 markedly reversed LPS‐induced down‐regulation of mHVEM (Fig. 1C) and up‐regulation of sHVEM (Fig. 1D). These data suggest that shedding of HVEM from monocytes could contribute to accumulation of circulatory sHVEM in patients with AH.

### Effect of Soluble HVEM on T‐Cell Functions

**Figure 2:**
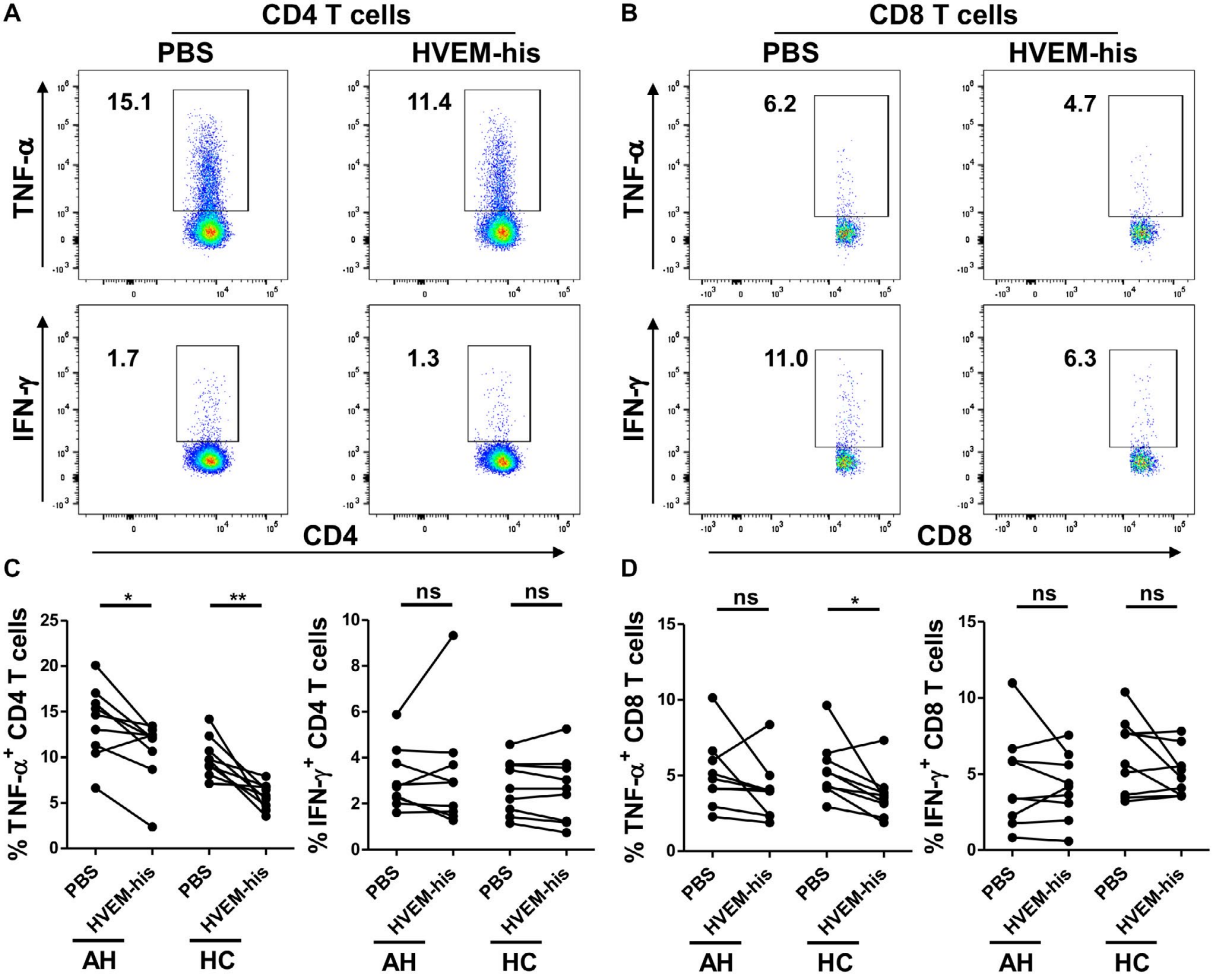
Effect of HVEM‐his on TNF‐α and IFN‐γ production by T cells from patients with AH and HCs. In A, B the box and number represent gate and percentage of cytokine positive T cells. In C,D each line shows data from an individual donor. (A,B) Representative plots and (C,D) summary graphs showing the effect of HVEM‐his on cytokine production by (A,C) CD4 and (B,D) CD8 T cells from patients with (A‐D) AH and (C,D) HCs. PBMCs were pretreated with HVEM‐his or PBS and stimulated with soluble anti‐CD3/anti‐CD28 antibodies (n = 9). Paired *t* test was performed to compare TNF‐α or IFN‐γ expression between HVEM‐his and PBS control; *P < 0.05, **P < 0.01.

To examine the function of sHVEM in AH, we assessed the effect of HVEM‐his on cytokine production by T cells in PBMCs from patients with AH versus HCs in response to T‐cell receptor (TCR) stimulation with anti‐CD3/anti‐CD28 antibodies. HVEM‐his consists of the soluble extracellular domain of human HVEM linked to a polyhistidine tag at the C‐terminus. HVEM‐his significantly inhibited TCR‐induced TNF‐α production by CD4 T cells from patients with AH and HCs (Fig. 2A,C). HVEM‐his also inhibited TNF‐α production by CD8 T cells from 8 out of the 9 patients with AH, although the difference did not reach significance (Fig. 2B,D). HVEM‐his did not significantly impact IFN‐γ production by T cells from patients with AH or HCs (Fig. 2).

**Figure 3:**
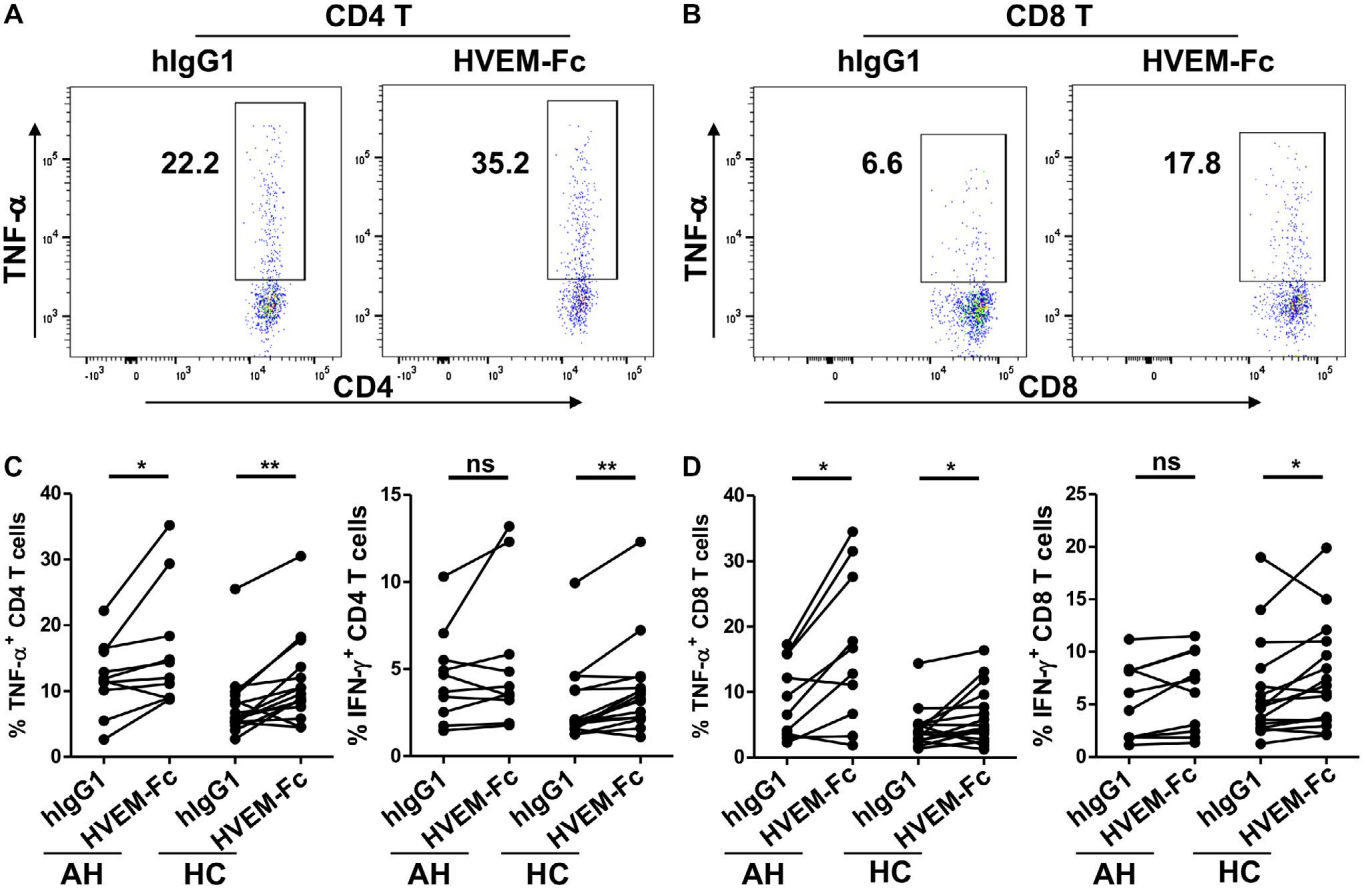
Effect of HVEM‐Fc on production of TNF‐α and IFN‐γ by T cells from patients with AH and HCs. In A, B the box and number represent gate and percentage of cytokine positive T cells. In C,D each line shows data from an individual donor. (A,B) Representative plots and (C,D) summary graphs showing the effect of recombinant HVEM‐Fc on production of TNF‐α and/or IFN‐γ by (A,C) CD4 and (B, D) CD8 T cells from patients with (A‐D) AH and (C,D) HCs. PBMCs from patients with AH or HCs were pretreated with HVEM‐Fc or human IgG1 isotype control and stimulated with anti‐CD3/anti‐CD28 antibodies (n = 10). Paired *t* test was performed to compare TNF‐α or IFN‐γ expression between HVEM‐Fc and hIgG1 control; *P < 0.05, **P < 0.01. Abbreviation: hIgG1, human IgG1 isotype control.

Soluble proteins are often manufactured as Fc‐fusion proteins due to the additional beneficial biological and pharmacologic properties of the immunoglobulin Fc domain, such as its ability to bind to the Fc receptors on immune cells. We next assessed the effect of HVEM‐Fc, which is composed of the extracellular domain of HVEM linked to the Fc region of a human IgG1, on TCR‐induced cytokine production. In contrast to HVEM‐his, HVEM‐Fc significantly enhanced TNF‐α production by CD4 and CD8 T cells from patients with AH and HCs (Fig. 3A‐D). Interestingly, HVEM‐Fc significantly enhanced IFN‐γ production by both CD4 and CD8 T cells from HCs but not from patients with AH (Fig. 3C,D), suggesting T cells from patients with AH are dysfunctional. Together, our data suggest that sHVEM plays an inhibitory role in HVEM network‐mediated TNF‐α production and that HVEM‐Fc likely acts as a membrane form through its interaction with the Fc receptors on monocytes, B cells, and NK cells to enhance T‐cell function either directly or indirectly.

## Discussion

In this study, we systematically profiled 18 sICPs in the plasma samples from a large cohort of patients with AH, HDCs, and HCs. In line with profound immune dysregulation in patients with AH,^3^, ^19^ we found that 17 out of 18 sICPs examined here were dysregulated (six up‐regulated and 11 down‐regulated). A previous study found that the TIM‐3 ligand galectin‐9 was elevated in patients with AH.^6^ Here, we identified six additional sICPs (sCD27, sCD40, sHVEM, sLIGHT, sTIM‐3, and sTLR‐2) that were up‐regulated in the blood of patients with AH. Interestingly, these six sICPs have been shown to be cleaved from membrane counterparts by sheddases.^23^, ^24^, ^25^ Consistent with shedding for the generation of these sICPs, we found that membrane forms of CD27, CD40, HVEM, and TIM‐3 were down‐regulated on the surface of subsets of blood immune cells in patients with AH.

We found that multiple MMPs, especially MMP7, were greatly elevated in patients with AH. In addition, MMP7 positively correlated with plasma levels of sCD27, sCD40, sHVEM, and sTLR‐2, whereas MMP2 levels correlated with sTIM‐3 levels in patients with AH, suggesting that they are involved in dysregulation of both soluble and membrane ICPs. It is possible that other sheddases also contributed to the cleavage of these molecules. MMP expression and activities are often enhanced by inflammatory cytokines, including IL‐8,^26^ which is one of the most highly up‐regulated inflammatory cytokines in patients with AH and is associated with disease severity.^19^, ^27^ Plasma levels of IL‐8 and MMP7/MMP2 correlated with levels of sCD27, sCD40, sHVEM, sTIM‐3, and sTLR‐2, suggesting that IL‐8 might contribute to production of these sICPs through up‐regulation of MMPs. Future study is warranted to verify this link.

Strikingly, 11 out of the 18 sICPs examined here were down‐regulated in patients with AH. Most of the 11 sICPs, including four inhibitory ICPs (BTLA, CTLA‐4, LAG‐3, and PD‐1) and three stimulatory ICPs (GITR, CD80, and ICOS), are generated through mRNA alternative splicing and secretion,^16^, ^28^, ^29^ whereas sCD28, sCD160, sPD‐L1 are cleaved from membrane‐bound counterparts.^23^, ^24^, ^25^ Consistent with their hyperactivated state, T cells from patients with AH expressed high levels of membrane‐bound inhibitory ICPs, including CTLA4, LAG‐3 and PD‐1. Thus, decreased production of sCTLA‐4, sLAG‐3, and sPD‐1 contrasted with up‐regulated expression of their membrane counterparts on T cells in patients with AH. The discorded expression of soluble versus membrane forms of CTLA‐4 is in line with a previous study.^18^ Alternatively spliced transcripts encoding sCTLA‐4 are the predominant CTLA‐4 transcripts in human resting T cells.^18^ Following T‐cell activation, full‐length CTLA‐4 transcripts encoding membrane CTLA‐4 increase while sCTLA‐4 transcripts decrease.^18^ On the other hand, T‐cell activation leads to a parallel increase in the expression of full‐length PD‐1 and sPD‐1 transcripts.^28^ Future analysis of the origin of sICPs in patients with AH will provide a better insight into mechanisms associated with their biosynthesis. The clinical significance of down‐regulation of sICPs needs further study.

Alcohol abstinence significantly improves disease outcome and survival but does not lead to complete recovery in most patients with AH.^30^ Consistent with this, we have recently reported that plasma levels of IL‐8 and TNF‐α are not normalized in abstinent patients with AH.^19^ Here, we identified four additional sICPs (sCD27, sCD40, sHVEM, and sTIM3) that were not fully reversed in abstinent patients with AH. Our results suggest that these sICPs could serve as sensitive markers for persistent immune activation in abstinent patients with AH. Strikingly, sCD27 and sCD40 levels were significantly higher in the deceased compared to the survivors. Elevated sCD40 in several liver diseases correlates with liver injury^31^ and could be used to detect liver injury in patients with chronic hepatitis B infection^.^ 32 Here, we also found that sCD40 could independently predict 90‐day mortality.

HVEM controls both inflammatory and inhibitory responses through its complex interactions with multiple stimulatory ligands and inhibitory receptors and bidirectional signaling.^8^, ^33^ Unlike many other ICPs, mHVEM is constitutively expressed on immune cells and nonhematopoietic cells.^7^ HVEM also exists as a soluble form in the circulation.^34^ Preclinical and clinical studies have suggested that dysregulation of the HVEM network contributes to multiple inflammatory and infectious diseases.^33^, ^35^, ^36^, ^37^ Currently, the expression pattern and roles of the HVEM axis in AH pathogenesis are unknown. Here, we found that expression of both soluble and membrane forms of the HVEM network were highly dysregulated in patients with AH. Soluble HVEM was among the highly up‐regulated sICPs in patients with AH. Studies have shown that circulating sHVEM is elevated in several allergic and autoimmune diseases^17^, ^34^ and in patients with hepatocellular carcinoma.^38^ We also found that mHVEM expression was down‐regulated on B cells and monocytes, suggesting those cells might be a source of sHVEM. A previous study found that patients with gastric cancer expressed less HVEM on leukocytes, including monocytes, and had higher levels of sHVEM.^17^ HVEM can be cleaved in an MMP‐dependent manner from human monocytes following stimulation by IL‐8, TNF‐α, and LPS.^17^ Consistent with this, we found that LPS and ethanol reduced mHVEM expression and increased sHVEM in an MMP‐dependent manner, suggesting that HVEM shedding from monocytes could contribute to sHVEM elevation in patients with AH. We also detected mHVEM on multiple human liver cell lines, including HepG2 and Hu7 hepatocytes (data not shown). Future study is needed to investigate whether mHVEM and sHVEM expression by these cells are affected by ethanol and/or inflammatory factors.

Our *ex vivo* study revealed that sHVEM inhibited TNF‐α production by T cells from patients with AH and HCs. Although it is not clear how sHVEM mediated this effect, it is possible that HVEM‐his interrupted the engagement of mHVEM on T cells by LIGHT on monocytes or neighboring activated T cells to down‐regulate HVEM‐mediated T‐cell costimulatory signaling.^7^, ^39^ Alternatively, sHVEM could act as an agonist to activate the inhibitory receptors BTLA/CD160 on T cells.^7^ Although clinical and experimental studies have shown that TNF‐α contributes to AH pathogenesis, TNF‐α neutralization in clinical trials failed to show any benefits for treatment of AH^40^, ^41^ and was actually associated with significantly higher rates of infections and mortality.^40^, ^41^ This is consistent with the notion that TNF‐α plays a critical role in antimicrobial defense. Thus, sHVEM dysregulation might contribute to enhanced susceptibility to infections in patients with AH. HVEM‐Fc might be used to reverse this defect in cytokine production by T cells through its binding to Fc receptors on immune cells, including APC, and acting as a membrane form to interact with its binding partners, such as LIGHT, on T cells.^7^, ^8^ As HVEM and its binding partners are expressed on different types of cells, sHVEM likely impacts the function of those cells in patients with AH. Future studies are needed to elucidate the mechanisms of action for sHVEM and the interplay between various soluble and membrane ICPs, including the HVEM axis. Furthermore, mechanistic studies using mice deficient of genes in the HVEM axis or by treatment with neutralizing antibodies will help to dissect the role of the HVEM pathways in AH pathogenesis.

In conclusion, we found that patients with AH had a broad dysregulation of sICPs in the peripheral circulation and that cessation of alcohol consumption greatly but not completely reversed these abnormalities. As circulatory sICP levels can be easily measured, they can be further explored as potential prognostic and/or predictive markers for AH. Development of therapies to target those dysregulated ICPs, including HVEM, may represent a potential strategy for restoring immune homeostasis and antimicrobial defense in patients with AH.
