# Supplementary figures and images for: Immune Checkpoint Axes Are Dysregulated in Patients With Alcoholic Hepatitis
Source: Hepatol Commun. 2020 Jan 12;4(4):588–605. doi: 10.1002/hep4.1475 (PMC7109345; doi:10.1002/hep4.1475)

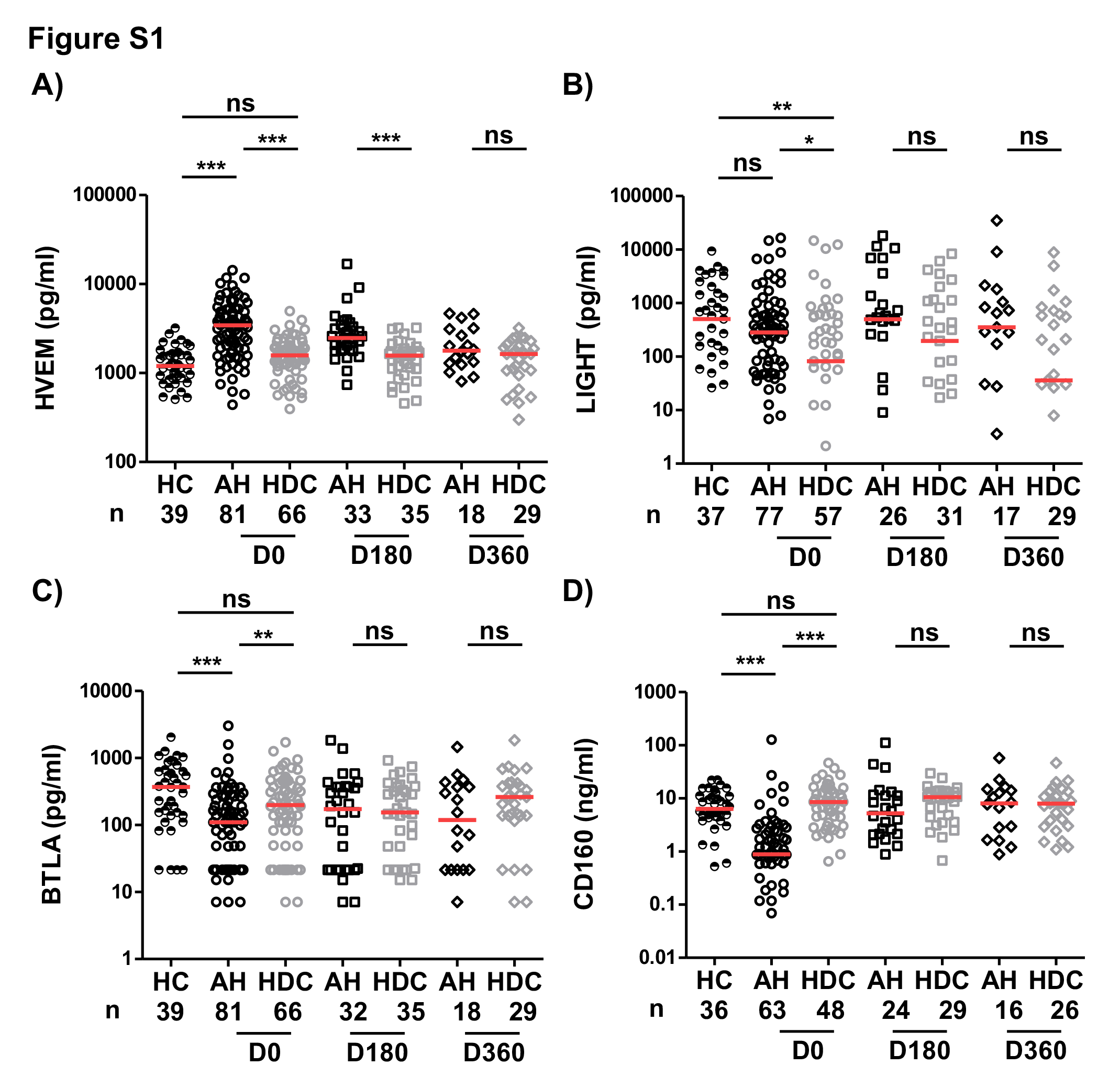

Supplement: Supplementary file 1 [file HEP4-4-588-s001.tif]

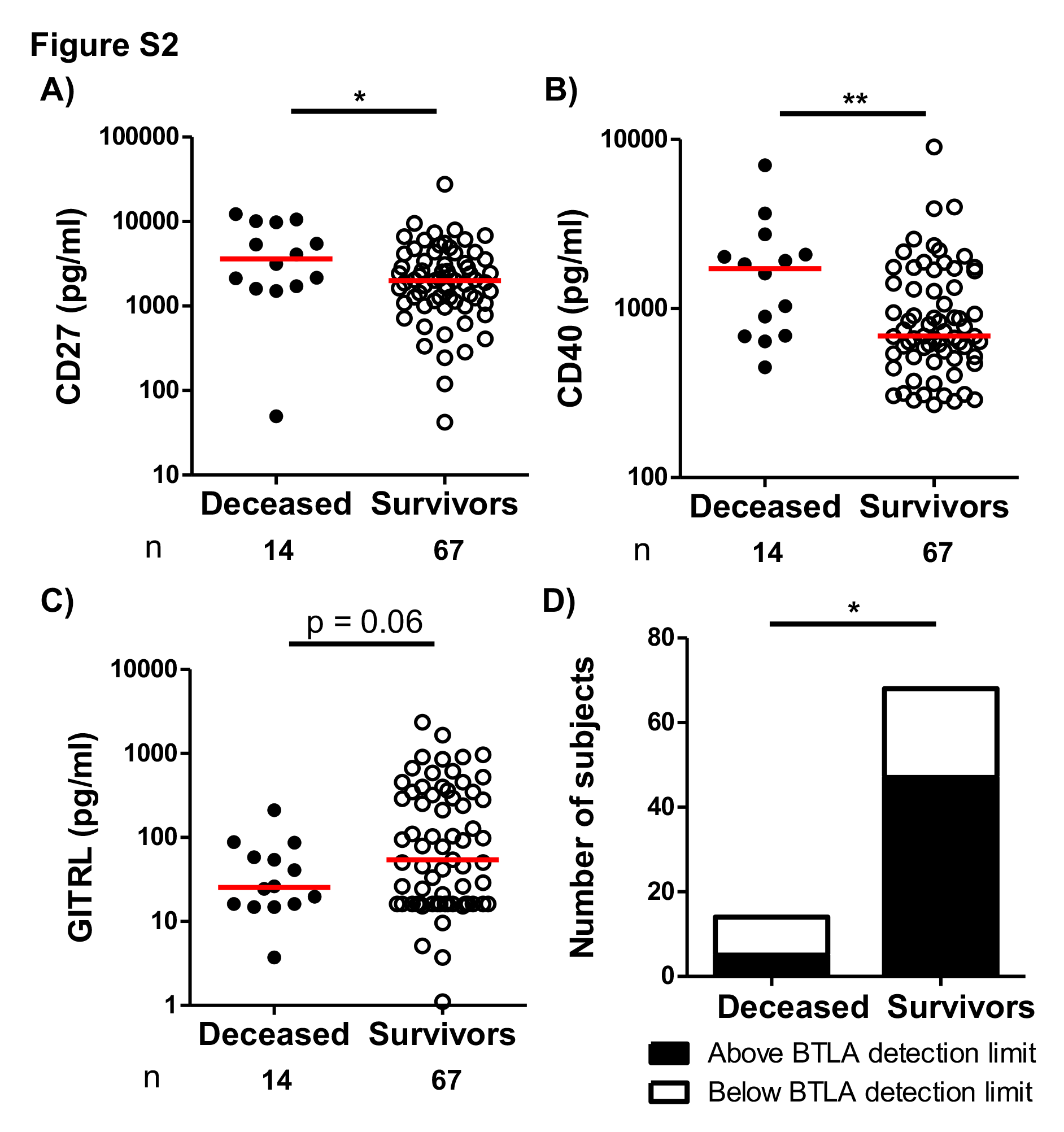

Supplement: Supplementary file 2 [file HEP4-4-588-s002.tif]

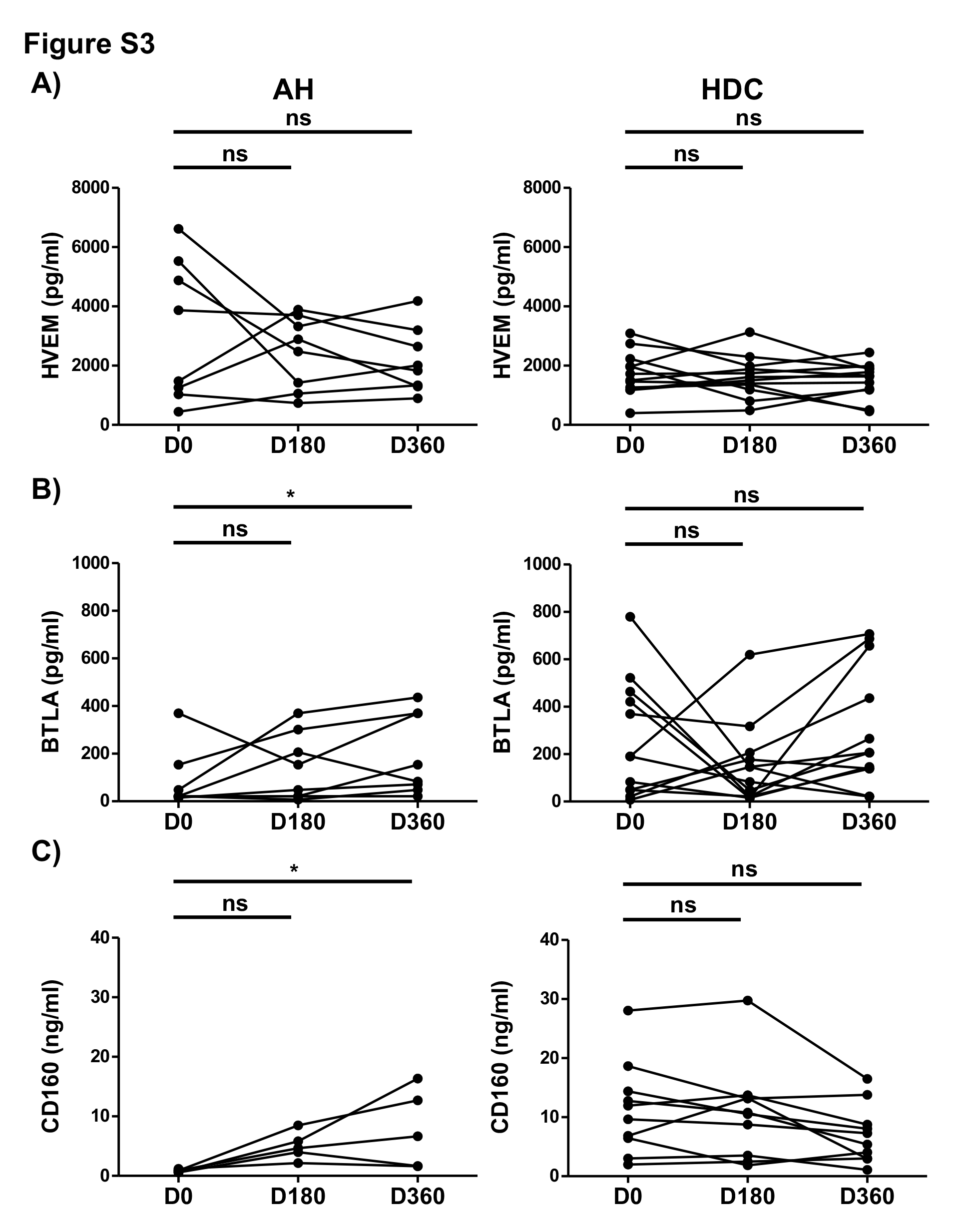

Supplement: Supplementary file 3 [file HEP4-4-588-s003.tif]

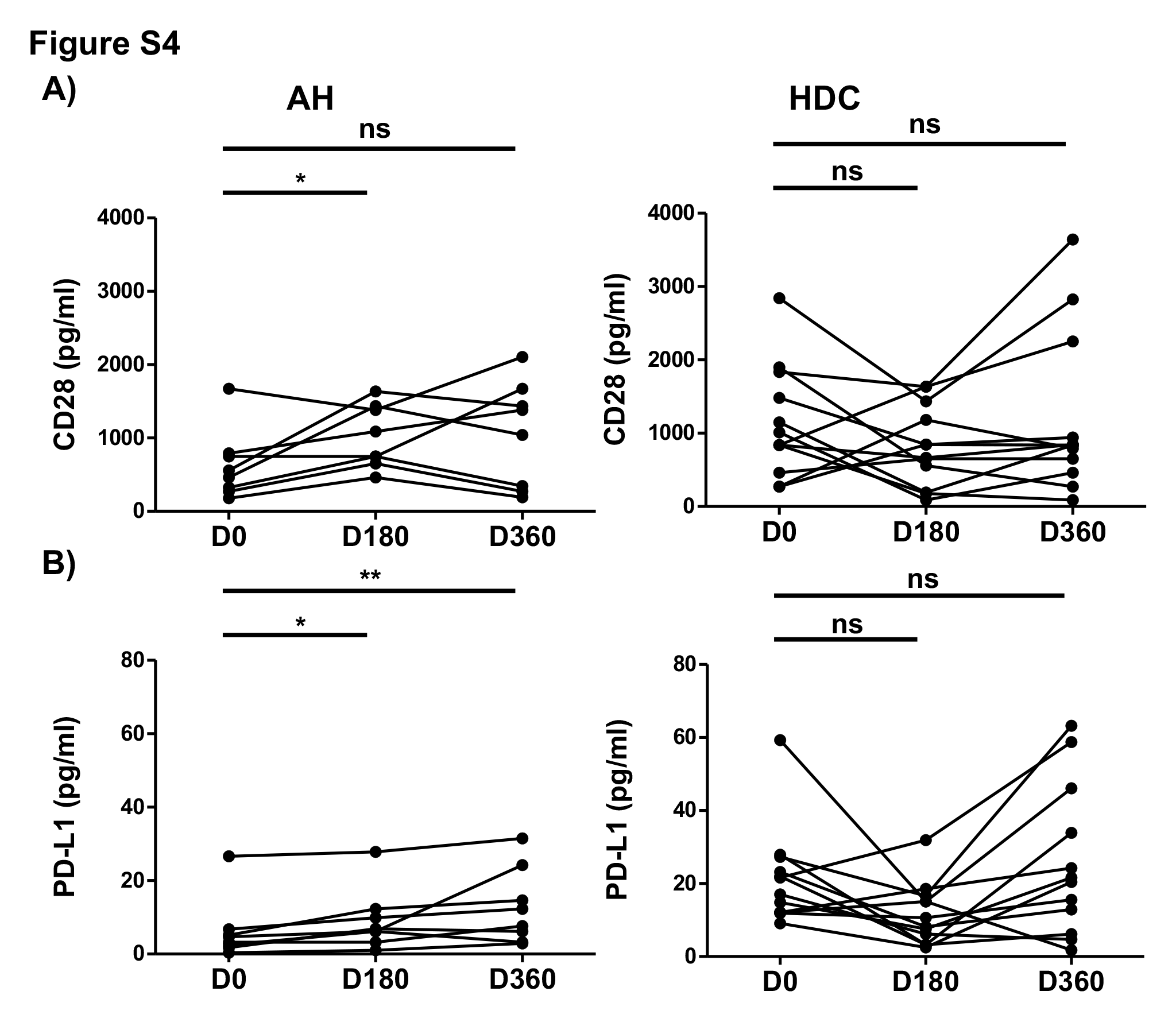

Supplement: Supplementary file 4 [file HEP4-4-588-s004.tif]

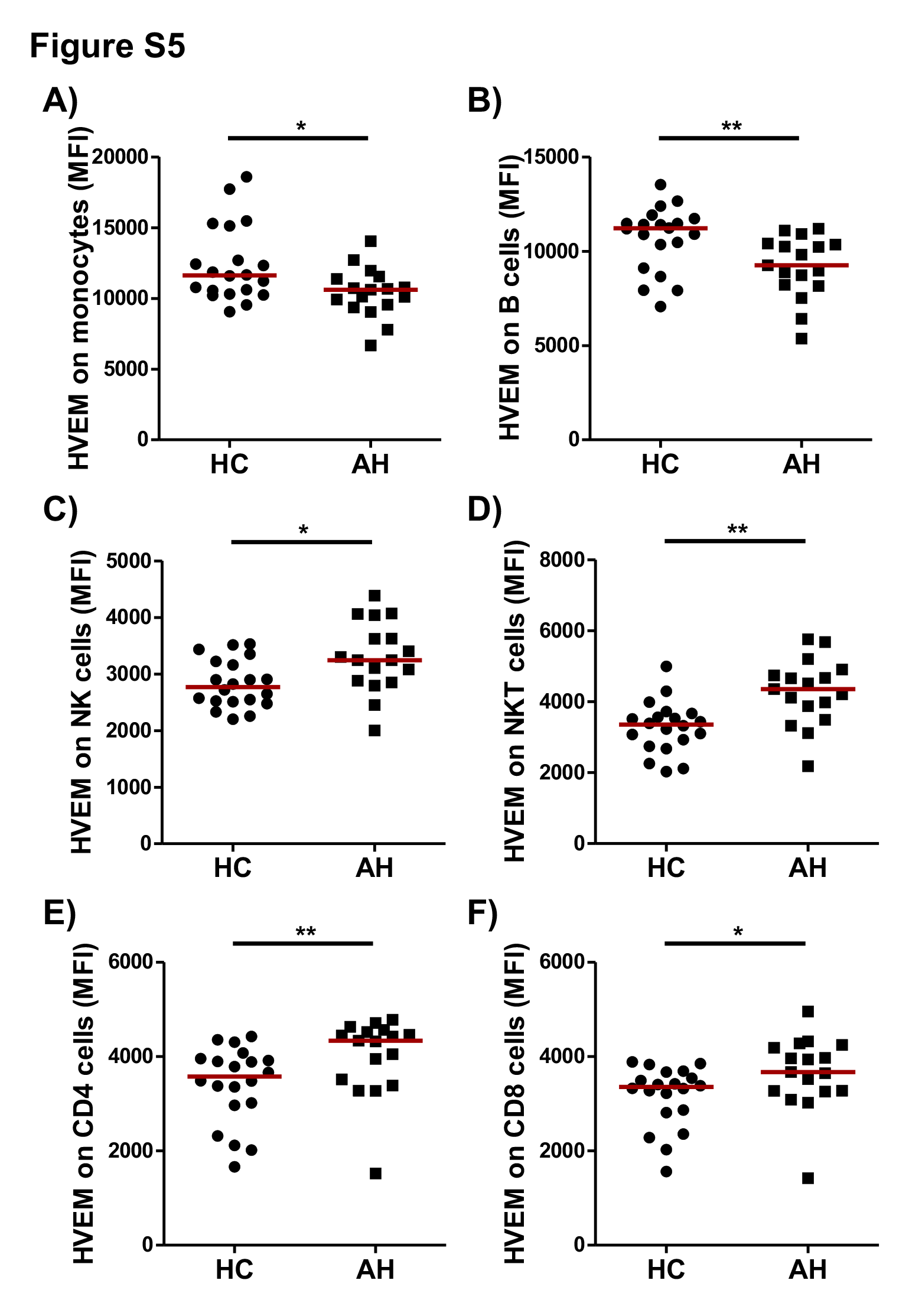

Supplement: Supplementary file 5 [file HEP4-4-588-s005.tif]
