# Supplementary material for: Immune Checkpoint Axes Are Dysregulated in Patients With Alcoholic Hepatitis
Source: Hepatol Commun. 2020 Jan 12;4(4):588–605. doi: 10.1002/hep4.1475 (PMC7109345; doi:10.1002/hep4.1475)
Supplement: Supplementary file 6 [file HEP4-4-588-s006.docx]

| Supporting Table S1. Characteristics of abstinent AH and HDC subjects at day 180 and day 360 follow-ups | | | | | | |
| --- | --- | --- | --- | --- | --- | --- |
| Variable | Day 180 | | | Day 360 | | |
|  | AH  (n=20) | HDC  (n=17) | *p* | AH  (n=14) | HDC  (n=13) | *p* |
| Age at enrollment  (years) | 45  (33-53) | 42  (32-49) | ns | 47  (38-54) | 44  (35-50) | ns |
| Gender  (% Male) | 65 | 76 | ns | 57 | 62 | ns |
| MELD score | 11  (8-17) | 7  (6-8) | *** | 10  (8-13) | 7  (6-8) | ** |
| Creatinine  (mg/dL) | 0.79  (0.66-1.06) | 0.9  (0.79-1.05) | ns | 1.01  (0.61-1.3) | 0.95  (0.87-1.06) | ns |
| Total bilirubin (mg/dL) | 1.55  (0.8-5.48) | 0.4  (0.3-0.45) | *** | 0.75  (0.58-1.33) | 0.4  (0.3-0.65) | ** |
| Prothrombin time (INR) | 1.29  (1.18-1.54) | 1.0  (0.96-1.06) | *** | 1.15  (1.05-1.3) | 1.02  (0.96-1.11) | * |
| AST (IU/L) | 33  (29-50) | 17  (16-23) | *** | 29  (25-35) | 20  (16-25) | ** |
| ALT (IU/L) | 25  (20-36) | 13  (11-24) | ** | 24  (19-28) | 17  (13-26) | ns |

**Note:** Data are shown as median and (interquartile ranges). AH patients: patients with alcoholic hepatitis; HDC: heavy drinking controls; Mann Whitney test and Chi-square test comparing AH patients versus HDC for continuous and categorical variables, respectively. **p* < 0.05, ***p* < 0.01, ****p* < 0.001, ns: not significant.

| Supporting Table S2. Fold changes in median plasma levels of soluble ICPs among AH, HDC, and HC subjects | | | | | | |
| --- | --- | --- | --- | --- | --- | --- |
| sICPs | | Day 0 | | | Day 180 | Day 360 |
|  |  | AH vs HC | AH vs HDC | HDC vs HC | AH vs HDC | AH vs HDC |
| Upregulated sICPs | TIM-3 | 5.2*** | 3.3*** | 1.6* | 1.9*** | 1.5** |
|  | HVEM | 2.9*** | 2.2*** | 1.3 | 1.6*** | 1.1 |
|  | CD40 | 2.7*** | 1.8*** | 1.5** | 1.4*** | 1.4 |
|  | CD27 | 2.4*** | 1.8*** | 1.3 | 1.9* | 1.2 |
|  | TLR-2 | 1.4 | 1.3* | 1.1 | 1.6* | 0.8 |
|  | LIGHT | 1.2 | 3.4* | 0.3** | 2.5 | 9.7 |
|  | CD86 | 1.0 | 1.1 | 0.9 | 1.5*** | 1.0 |
| Downregulated  sICPs | PD-1 | 0.7 | 0.6* | 1.1 | 1.1 | 0.9 |
|  | CD28 | 0.6** | 0.5*** | 1.2 | 0.8 | 1.0 |
|  | PD-L1 | 0.5*** | 0.4*** | 1.1 | 0.9 | 0.2** |
|  | LAG-3 | 0.5*** | 0.5*** | 0.9 | 1.2 | 0.8 |
|  | CD80 | 0.4*** | 0.6*** | 0.6* | 1.0 | 0.8 |
|  | GITR | 0.4** | 1.0* | 0.4 | 0.6 | 0.6 |
|  | CTLA-4 | 0.3*** | 0.4*** | 0.9 | 0.8 | 0.3* |
|  | BTLA | 0.3*** | 0.5** | 0.5 | 0.5 | 1.1 |
|  | GITRL | 0.3*** | 0.4 | 0.6* | 1.3 | 1.1 |
|  | CD160 | 0.2*** | 0.1*** | 1.5 | 0.5 | 1.0 |
|  | ICOS | 0.1*** | 0.3*** | 0.4 | 0.4 | 0.3 |

**Note:** AH patients: patients with alcoholic hepatitis; HDC: heavy drinking controls; HC: healthy controls. Kruskal-Wallis test with Dunn’s correction for pairwise comparisons among AH patients, HDC, and HC at baseline (Day 0). Mann Whitney test comparing AH patients versus HDC at day 180 and day 360. D0: AH patients n=63-81, HDC n=48-65; D180: AH patients n=24-33, HDC n=29-32; D360: AH patients n=16-18, HDC n=26-29; HC n=34-39. **p* < 0.05, ***p* < 0.01, ****p* < 0.001.

| Supporting Table S3. Univariate and multivariate Cox regression analysis of 90-day mortality | | | | | | | |  |
| --- | --- | --- | --- | --- | --- | --- | --- | --- |
| Variables | | Univariate Cox regression | | | Multivariate Cox regression | | |  |
|  |  | HR | 95% CI | *p* | HR | 95% CI | *p* | |
| Clinical and demographic parameters | Age | 1.002 | 0.95-1.056 | 0.949 |  |  |  | |
|  | Gender | 0.709 | 0.238-2.116 | 0.538 |  |  |  | |
|  | mDF | 1.015 | 1.003-1.028 | 0.011 | 1.003 | 0.986-1.02 | 0.757 | |
|  | MELD | 1.064 | 1.005-1.125 | 0.032 | 0.975 | 0.853-1.115 | 0.715 | |
|  | ALT | 1.004 | 0.989-1.018 | 0.623 |  |  |  | |
|  | AST | 0.998 | 0.989-1.007 | 0.655 |  |  |  | |
|  | CRP | 1.024 | 1.005-1.043 | 0.013 | 1.01 | 0.987-1.033 | 0.409 | |
|  | Treatment | 3.027 | 0.844-10.851 | 0.089 | 1.887 | 0.397-8.97 | 0.425 | |
| Upregulated sICPs | TIM3 | 1 | 1-1 | 0.086 | 1 | 1-1 | 0.973 | |
|  | HVEM | 1 | 1-1 | 0.265 |  |  |  | |
|  | CD40 | 1 | 1-1 | 0.025 | 1.000 | 1-1.001 | 0.014 | |
|  | CD27 | 1 | 1-1 | 0.08 | 1 | 1-1 | 0.539 | |
|  | TLR2 | 1 | 1-1 | 0.069 | 1 | 1-1 | 0.916 | |
|  | LIGHT | 1 | 1-1 | 0.561 |  |  |  | |
| Downregulated sICPs | PD-1 | 1 | 1-1 | 0.299 |  |  |  | |
|  | CD28 | 1 | 1-1.001 | 0.05 | 1.000 | 1-1.001 | 0.033 | |
|  | PD-L1 | 1.008 | 0.978-1.038 | 0.607 |  |  |  | |
|  | LAG-3 | 1 | 1-1 | 0.705 |  |  |  | |
|  | CD80 | 0.996 | 0.959-1.034 | 0.825 |  |  |  | |
|  | GITR | 0.96 | 0.867-1.064 | 0.441 |  |  |  | |
|  | CTLA-4 | 0.97 | 0.919-1.024 | 0.271 |  |  |  | |
|  | BTLA | 0.999 | 0.997-1.002 | 0.633 |  |  |  | |
|  | GITRL | 0.994 | 0.987-1.001 | 0.093 | 0.994 | 0.983-1.006 | 0.348 | |
|  | CD160 | 0.521 | 0.243-1.117 | 0.094 | 0.366 | 0.151-0.889 | 0.026 | |
|  | ICOS | 1.001 | 0.998-1.003 | 0.651 |  |  |  | |

**Note:** HR, hazard ratio; mDF, Maddrey’s discriminant function; MELD, model for end-stage liver disease; ALT, alanine aminotransferase; AST, aspartate aminotransferase; CRP, c-reactive protein; Treatment, patients treated with or without corticosteroids and/or pentoxifylline at study entry.

| Supporting Table S4. Differences in clinical parameters and median levels of soluble ICPs between AH Patients Treated with and without Corticosteroids and/or pentoxifylline | | | | | | | | |
| --- | --- | --- | --- | --- | --- | --- | --- | --- |
| Variables | | Day 0 | | Day 180 | | Day 360 | |  |
|  |  | Treated | Non-treated | Treated | Non-treated | Treated | Non-treated |  |
| Clinical parameters | mDF score | 46**  (22-72) | 22  (8-45) | 38  (6-53) | 18  (-2-32) | 40  (32-87) | 24  (6-30) |  |
|  | MELD score | 26**  (22-28) | 22  (15-23) | 9  (8-21) | 11  (8-18) | 8  (7-13) | 10  (10-13) |  |
|  | Creatinine (mg/dL) | 0.8  (0.6-1.3) | 0.7  (0.6-1.0) | 0.8  (0.7-1.0) | 0.8  (0.6-1.2) | 1.0  (0.6-1.1) | 1.2  (0.6-1.4) |  |
|  | Total bilirubin  (mg/dL) | 16.2***  (10.6-25.8) | 8.3  (4.9-15.3) | 1.2  (0.7-6.9) | 1.6  (0.8-6.3) | 0.7  (0.4-1.8) | 0.8  (0.7-1.3) |  |
|  | Prothrombin time (INR) | 2.0***  (1.6-2.3) | 1.53  (1.3-1.8) | 1.3  (1.2-2.1) | 1.3  (1.1-1.5) | 1.1  (1.1-1.5) | 1.2  (1-1.2) |  |
|  | AST  (IU/L) | 106  (82-139) | 115  (90-170) | 32  (29-47) | 35  (29-66) | 28  (23-34) | 29  (26-35) |  |
|  | ALT  (IU/L) | 44  (31-59) | 47  (25-63) | 20*  (15.5-24) | 27  (23-50) | 23  (15-32) | 24  (20-26) |  |
| Up-regulated sICPs  (pg/ml) | TIM-3 | 7298*  (4557-11028) | 4652  (2650-8386) | 4351**  (3753-4787) | 3196  (1663-4046) | 2662  (1817-5060) | 3449  (2061-4744) |  |
|  | HVEM | 3795  (2115-5108) | 2356  (1357-5067) | 3150*  (2742-5652) | 2282  (1424-3325) | 2011  (1293-3196) | 2044  (1338-4184) |  |
|  | CD40 | 937*  (637-1755) | 634  (388-890) | 556  (507-755) | 535  (439-642) | 479  (359-547) | 508  (359-700) |  |
|  | CD27 | 2140  (1496-4327) | 2007  (1141-3724) | 2795*  (1719-7806) | 1781  (896-3051) | 1709  (1236-2595) | 1860  (1002-5158) |  |
|  | TLR-2 | 732  (457-916) | 520  (364-837) | 491  (321-555) | 360  (232-923) | 376  (196-829) | 414  (176-2063) |  |
|  | LIGHT | 302  (56-656) | 269  (41-697) | 491  (120-2064) | 618  (18-3609) | 283  (28-1054) | 510  (3-3643) |  |
| Down-regulated sICPs  (pg/ml) | PD-1 | 290  (178-421) | 243  (163-395) | 349  (248-616) | 269  (242-556) | 374  (306-598) | 349  (199-1139) |  |
|  | CD28 | 666  (204-1094) | 653  (192-1163) | 750  (495-1310) | 1087  (653-1671) | 847  (344-1671) | 1380  (272-2106) |  |
|  | PD-L1 | 10  (5-16) | 8  (4-12) | 10  (6-16) | 10  (5-27) | 6  (3-24) | 8  (3-21) |  |
|  | LAG-3 | 1665  (1042-2615) | 1332  (799-3386) | 1989  (1514-2771) | 3231  (844-6239) | 2251  (1376-3314) | 1727  (844-5833) |  |
|  | CD80 | 8  (4-9) | 9  (5-11) | 9  (6-11) | 9  (9-21) | 9  (5-18) | 9  (5-24) |  |
|  | GITR | 6.3*  (6.2-6.3) | 6.3  (6.3-6.3) | 6.3  (6.3-17.8) | 6.3  (6.1-21.5) | 10.5  (6.3-27.1) | 10.5  (6.3-37.1) |  |
|  | CTLA-4 | 10  (4-18) | 11  (4-22) | 7  (4-26) | 17  (11-37) | 17  (9-23) | 23  (3-66) |  |
|  | BTLA | 99  (22-191) | 139  (22-269) | 48  (22-292) | 192  (22-376) | 154  (71-436) | 48  (22-370) |  |
|  | GITRL | 24**  (16-101) | 103  (26-427) | 54  (16-191) | 320  (21-669) | 239  (54-435) | 67  (31-722) |  |
|  | CD160 | 884**  (640-1557) | 1789  (1043-3581) | 8468  (2116-13032) | 4663  (1685-38286) | 6664  (1207-14041) | 11675  (2522-26601) |  |
|  | ICOS | 45  (33-72) | 45  (45-70) | 57  (45-123) | 45  (45-333) | 95  (45-160) | 52  (45-333) |  |

**Note:** Data are shown as median and (interquartile ranges). ICPs, immune checkpoints; AH, patients with alcoholic hepatitis; Treated, patients treated with corticosteroids and/or pentoxifylline at study entry; Non-treated, AH patients not treated with the drugs; mDF, Maddrey discriminant function; MELD, model for end-stage liver disease; AST, aspartate aminotransferase; ALT, alanine aminotransferase; INR, international normalized ratio; s. Day 180 and Day 360 samples were from AH patients who were abstinent at follow up. Mann Whitney test comparing treated versus untreated AH patients at baseline (Day 0), day 180 and day 360. Day 0: Treated n=37-48, NT n=21-33; Day 180: Treated n=9, NT n=6-11; Day 360: Treated n=7, NT n=6-7. **p* < 0.05, ***p* < 0.01, ****p* < 0.001.

| **Supporting Table S5. Comparison of plasma levels of bacterial translocation (BT)-associated markers and MMPs** | | | | | | | | |
| --- | --- | --- | --- | --- | --- | --- | --- | --- |
| Factors  (ng/ml) | | Day 0 (baseline) | | | Day 180 | | Day 360 | |
|  |  | HC  (n=24-37) | AH  (n=61-87) | HDC  (n=55-66) | AH  (n=30-31) | HDC  (n=24-32) | AH  (n=16-19) | HDC  (n=22-28) |
| BT-associated markers | LBP (μg/ml) | 6.3^###^  (5.2-8.1) | 12.7***  (7.8-16.9) | 8.4  (6.4-10.78) | 8.15  (6.7-10) | 6.85  (4.85-10) | 6.55  (4.4-9.1) | 7.4  (4.6-11.3) |
|  | LPS (EU/ml) | 0.01^###^  (0.01-0.01) | 0.02***  (0.01-0.608) | 0.01  (0.01-0.01) | 0.01  (0.01-0.29) | 0.01  (0.01-0.03) | 0.01  (0.01-0.04) | 0.03  (0.01-0.07) |
|  | sCD14 | 1186^###^  (999-1590) | 2139***  (1650-2871) | 1541^§^  (1261-1925) | 1636*  (1278-2070) | 1227  (1135-1556) | 1729  (1354-2034) | 1371  (1116-1657) |
|  | sCD163 | 546^###^  (390-720) | 4146***  (2803-5371) | 524  (371-759) | 1245***  (848.8-1932) | 463  (359.8-628.5) | 996***  (867-1671) | 636  (402-853) |
| MMPs | MMP-1 | 1.145^###^  (0.79-1.828) | 5.495**  (2.863-9.665) | 3.35^§§§^  (1.833-5.635) | 3.91  (1.57-5.58) | 1.91  (0.94-2.96) | 3.01  (1.8-3.9) | 2.08  (1.51-3.47) |
|  | MMP-2 | 158^###^  (40.5-202.6) | 344.6***  (289-438) | 188.9  (168.5-213.3) | 309.2***  (219.6-400.7) | 177.4  (125.8-201.3) | 270***  (237-378) | 175  (148-196) |
|  | MMP-7 | 17^###^  (13.64-22.8) | 97.63***  (48.35-183.8) | 17.35  (13.41-21.99) | 45.41***  (26.39-93.63) | 14.68  (12.47-18.64) | 58***  (30-98) | 16.7  (13.6-21.9) |
|  | MMP-9 | 44.85^###^  (5.598-76.3) | 143.2  (74.8-272.4) | 140.9^§§§^  (93.69-248.7) | 124.4  (67.98-190.8) | 126.9  (80.77-240.3) | 104.8  (49.36-201.1) | 125.4  (75.1-172.1) |
|  | MMP-10 | 0.31^###^  (0.21-0.6) | 0.8***  (0.41-1.48) | 0.4  (0.21-0.45) | 0.7**  (0.31-0.99) | 0.31  (0.11-0.45) | 0.56*  (0.41-1.35) | 0.4  (0.2-0.6) |

Note: Data are represented as median and (interquartile ranges) in ng/ml unless stated otherwise. BT, bacterial translocation; MMPs, matrix metalloproteinases (MMPs). Kruskal-Wallis test with Dunn’s correction for pairwise comparisons among HC, AH patients, and HDC at enrollment (Day 0). Mann Whitney test comparing AH patients versus HDC at day 180 and day 360 follow-up. ^###^*p* < 0.001 for comparison between AH patients and HC at Day 0; **p* < 0.05, ***p* < 0.01, ****p* < 0.001 for comparison between AH patients and HDC; ^§^*p* < 0.05, ^§§§^*p* < 0.001 for comparison between HDC and HC at Day 0.

| **Supporting Table S6. Fold changes in median plasma levels of bacterial translocation markers and MMPs among AH, HDC, and HC subjects** | | | | | | |
| --- | --- | --- | --- | --- | --- | --- |
| Variables | | Day 0 | | | Day 180 | Day 360 |
|  |  | AH vs HC | AH vs HDC | HDC vs HC | AH vs HDC | AH vs HDC |
| BT markers | LPS | 2.0*** | 2.0*** | 1.0 | 1.2 | 0.3 |
|  | LBP | 2.0*** | 1.5*** | 1.3 | 1.2 | 0.9 |
|  | sCD14 | 1.8*** | 1.4*** | 1.3* | 1.3* | 1.3 |
|  | sCD163 | 7.6*** | 7.9*** | 1.0 | 2.7*** | 1.6*** |
| MMPs | MMP-1 | 4.8*** | 1.6** | 2.9*** | 2.0 | 1.5 |
|  | MMP-2 | 2.2*** | 1.8*** | 1.2 | 1.7*** | 1.5*** |
|  | MMP-7 | 5.7*** | 5.6*** | 1.0 | 3.1*** | 3.5*** |
|  | MMP-9 | 3.2*** | 1.0 | 3.1*** | 1.0 | 0.8 |
|  | MMP-10 | 2.6*** | 2.0*** | 1.3 | 2.3** | 1.4* |

**Note:** BT: bacterial translocation; AH patients: patients with alcoholic hepatitis; HDC: heavy drinking controls; HC: healthy controls. Kruskal-Wallis test with Dunn’s correction for pairwise comparisons among AH patients, HDC, and HC at baseline (Day 0). Mann Whitney test comparing AH patients versus HDC at day 180 and day 360 follow-ups. **p* < 0.05, ***p* < 0.01, ****p* < 0.001. D0: AH patients n=61-87, HDC n=55-66; D180: AH patients n=30-31, HDC n=24-32; D360: AH patients n=16-19, HDC n=22-28; HC n=24-37.

| **Supporting Table S7. Fold changes in expression levels of membrane ICPs on subsets of peripheral blood immune cells between AH and HC subjects** | | | | | | | | | | | | |
| --- | --- | --- | --- | --- | --- | --- | --- | --- | --- | --- | --- | --- |
| Varibles | | Exhaustion/inhibitory ICPs | | | | Stimulatory ICPs | | HVEM axis | | | | |
|  |  | CTLA-4 | LAG-3 | PD-1 | TIM-3 | CD27 | CD40 | HVEM | BTLA | CD160 | LIGHT |  |
| APCs | Monocytes | 1.28* | 1.15** | 0.53*** | 0.67** | 1.02 | 1.19 | 0.91* | 1.47* | 1.06 | 1.20 |  |
|  | B cells | 0.87* | 1.27 | 1.00 | nd | 0.87 | 0.82** | 0.83** | 0.76* | 0.61** | 1.18 |  |
| LYs | NK cells | 1.09 | 1.20 | nd | 0.91 | 1.00 | 1.23 | 1.17* | 1.81*** | 0.64*** | 1.11 |  |
|  | NKT cells | 1.15 | 1.26* | 0.88 | 1.28* | 1.33 | 1.22 | 1.30** | 1.18 | 0.77 | 0.75 |  |
|  | CD4 T cells | 1.32** | 1.23*** | 1.39* | 1.19* | 0.78* | 1.01 | 1.21** | 0.88 | 0.96 | 0.47 |  |
|  | CD8 T cells | 1.16 | 1.46* | 1.10 | 1.29*** | 1.29 | 1.14 | 1.09* | 0.98 | 0.73 | 1.23 |  |

**Note:** Data are presented as fold change in geometric mean fluorescent intensity (MFI) between patients with alcoholic hepatitis (AH) and healthy controls (HC). ICPs: immune checkpoints; APCs: antigen-presenting cells; LYs: lymphocytes. Mann Whitney test was used to compare MFI levels of ICPs expressed on peripheral blood cells of AH patients versus HC. **p* < 0.05, ***p* < 0.01, ****p* < 0.001. For ICPs except PD-1 and LIGHT, n=15-17 for AH patients; n=16-20 for HC. For PD-1, n=24 for AH patients; n=30 for HC. For LIGHT, n=8 for AH patients; n=11 for HC.
